# Making smartglasses accessible: perspectives and prototypes from co-design with people with aphasia

**DOI:** 10.1038/s41598-025-22253-2

**Published:** 2025-11-03

**Authors:** Humphrey Curtis, Timothy Neate

**Affiliations:** Department of Informatics, King’s College London, London, WC2B 4BG United Kingdom

**Keywords:** Computer science, Information technology

## Abstract

Smartglasses are set to become a mainstream consumer technology in the near future. However, emerging technologies often overlook the accessibility needs of users with disabilities and older adults, increasing the risk of alienation and marginalization of these vulnerable communities. To address this tension, we conducted three co-design workshops with people living with aphasia (N=14) to examine their perspectives on smartglasses, envision potential applications, and identify anticipated barriers. Co-designers proposed and prototyped a variety of smartglass applications for both general and aphasia-specific support. However, co-designers also raised concerns about interaction difficulties and the socially conspicuous form-factor of current smartglass designs. Additionally, we evaluated smartglass functionalities using a mixed reality HoloLens head-mounted display (HMD). While participants expressed enthusiasm, qualitative and quantitative findings revealed mixed reactions. The standard hands-free interaction of the HoloLens was perceived as publicly awkward and deemed inaccessible for participants with vision impairments or post-stroke paralysis. Our findings highlight the need for more inclusive design practices to ensure emerging smartglasses empower all users.

## Introduction

Over the past decade, mixed reality (MR) smartglasses and headsets have steadily emerged as consumer technologies^1,2^. Recent product launches have been positioned as breakthrough devices – described by their developers as the *“most advanced consumer device ever developed”* and even a potential *“replacement for the smartphone”*^3^. Smartglasses are formally defined as wearable technology that enables users to superimpose digital information onto the physical world in real time through augmented reality (AR) overlays, heads-up displays (HUDs), and head-mounted displays (HMDs)^4^. To seamlessly support this advanced technological interaction, smartglasses usually incorporate several displays, built-in cameras, microphones, speakers, touch-sensitive controls, and wireless connectivity. However, these hardware demands often result in bulky, heavy, and socially conspicuous headwear^4^.

**Fig. 1:**
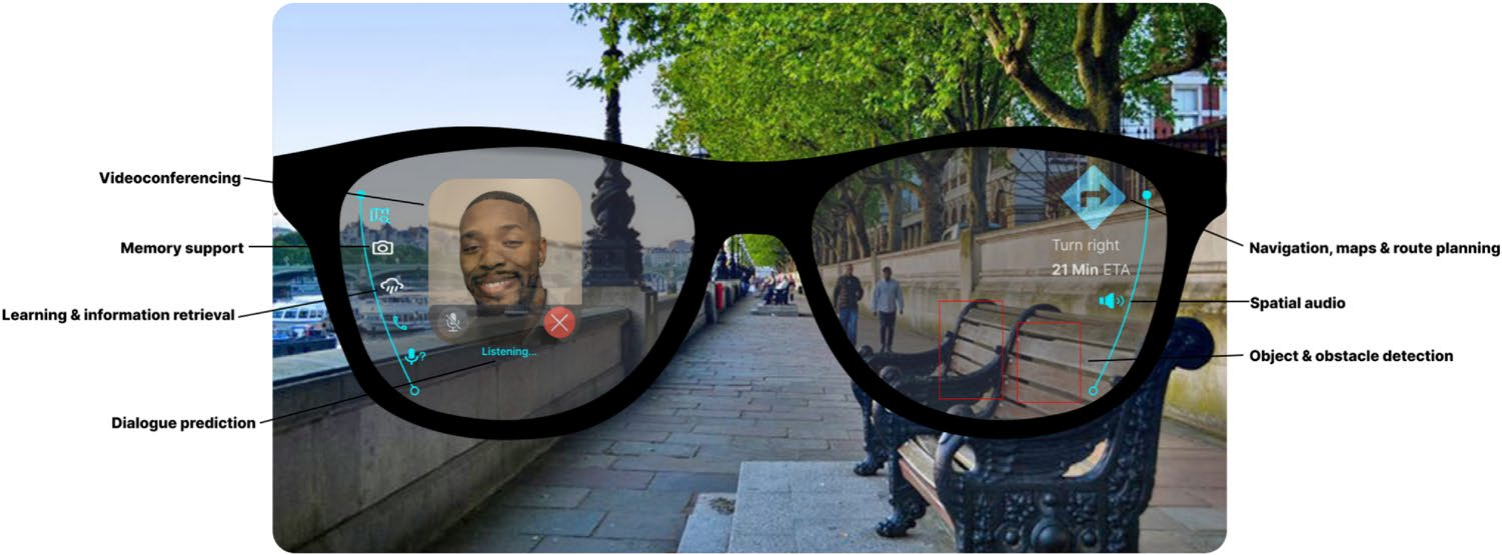
Envisioned smartglass interface for public navigation and communication for a user with aphasia in London. The user interface elements and prototype were created by the first author based on co-designers’ recommendations from three co-design workshops. The figure was developed using open-access assets from the Figma Community, which are published under licenses that permit reuse and adaptation without copyright restrictions. No copyrighted material requiring permission has been used.

With smartglasses poised for mainstream adoption, it is crucial to explore their assistive potential and general accessibility for older adults and people living with disabilities. Too often, emerging technologies remain inaccessible, systematically marginalizing many vulnerable groups and communities^5,6^. In contrast, traditional corrective eyewear presents a compelling example of a socially accepted and mainstream assistive technology^7,8^. Unlike other assistive technologies (e.g., canes, hearing aids) glasses with corrective lenses have been found to avoid equivalent social stigmas^8–10^. Instead, glasses function as both vision aids and fashionable accessories, offering wearers a wide range of designs, frame shapes, materials, and weights to ensure desirability, customization, and style^7^.

Building on this promising foundation, we employ participatory design, to co-design assistive smartglasses for adults living with aphasia. Co-design actively involves all stakeholders and end-users in the design process to ensure the technology aligns with their needs and expectations^11,12^. Meanwhile aphasia is a language impairment that can occur after a stroke, resulting in varied challenges with language formulation, production, and comprehension^13^. Approximately one-third of stroke survivors experience aphasia, affecting around 2 million people in the USA and 0.5 million in the UK^13^. The prevalence of aphasia is expected to rise significantly in the coming years due to an ageing global population and increased stroke survival rates^13^. Beyond language difficulties, people with aphasia contend with a multitude of life-limiting physical, social, mobility, and age-related challenges including: bodily paralysis, vision impairments^14^, social isolation, identity issues^15^, and difficulties with independent navigation^16,17^.

To address these difficulties, people with aphasia are often prescribed a range of low and high-tech assistive technologies including: prescription glasses, walking sticks, arm/leg braces, communication devices (see^18^ for a systematic review), speech therapy apps^19^, text-to-speech (TTS) and therapy^20^. Specifically for language difficulties, people with aphasia can be trained to use augmentative and alternative communication (AAC) devices to support their daily communication needs^18,21,22^. High-tech AAC devices and apps generate speech via electronic speech synthesis to assist with communication^18,23,24^. More generally, smartphones have been found to greatly support people with aphasia’s independence – navigation apps enable mobility, whilst texts, phone and video calls ensure remote communication with friends/family and healthcare^25,26^.

Although assistive technologies hold immense promise for many people living with aphasia, currently their long-term adoption rates are disappointingly low – with eventual abandonment alarmingly common^24,27^. Research has identified several reasons, including that assistive technologies can contribute to: social stigma, lack intuitiveness, and require extensive training^10,24,27,28^. For example, tablet-based AAC devices often require slow, two-handed interaction, which can hinder people with aphasia by limiting their existing mobility and residual verbal/non-verbal communication abilities^26,29^. In contrast, looking to the future, wearable smartglasses – such as those depicted in Fig. 1 – could support people with aphasia more intuitively and discreetly *‘on the go’*, empowering independence, mobility and communication without debilitating social stigmas^8^. However, to date, no research has sought to co-design assistive smartglasses in direct collaboration with communities living with aphasia^12^. Consequently, in this research we address these prominent research gaps by:

- Exploring people with aphasia’s perspectives on MR smartglasses, envisaged applications and anticipated barriers.
- Present workshop findings from exploring smartglass functionalities through a high-fidelity MR HoloLens HMD prototype, yielding notable accessibility insights for future smartglass research and development.

## Related work

Emerging research has begun to explore the potential of smartglasses and HMDs as wearable, unobtrusive assistive technologies across several disabled communities. To begin, Creed et al. conducted two broad workshops on inclusive augmented reality (AR) and virtual reality (VR) experiences, identifying accessibility barriers encountered by a range of disabled communities^30^. In contrast, Bae et al. systematically reviewed 9 studies focused on MR technologies for people with visual impairments^31^ – they found that most existing research has predominantly concentrated on MR applications for medical rehabilitation and training, primarily aimed at improving users mobility and autonomy.

Beyond people visual impairments, smartglasses and VR technologies have largely been applied to support safe learning, rehabilitation and therapy for communities with disabilities. For example, Aruanno et al. developed HoloLearn, a Hololens application designed to support 20 individuals with neuro-developmental disorders in performing everyday tasks, with the aim of fostering greater independence in domestic settings^32^. A follow-up study expanded HoloLearn to include a speech-based virtual assistant to support the rehabilitation of 15 people with cognitive disabilities^33^. Similarly, Imashev et al. piloted a Hololens application to support 19 deaf and hard-of-hearing (DHH) children in acquiring sign language^34^. Finally, VR technologies have also been used to optimise motor-function learning amongst people with Parkinson’s Disease (PD)^35^.

Extending these prior studies, our research presents findings from co-design workshops conducted directly with people living with aphasia. Moving beyond a purely medical or rehabilitative focus, we aim to foreground the diverse perspectives of people with aphasia regarding smartglasses – their wider perspectives, envisioned applications and anticipated barriers.

## Methods

We conducted three interconnected MR smartglass co-design workshops with 14 people living with aphasia and 7 speech and language therapists (SLTs). The GRIPP2 Short Form, documenting patient engagement in the research, is available in the supplementary materials (SM1) – this was completed in line with best practices to enhance transparency, reproducibility and alignment.

### Research environment and procedures

This research was conducted within the context of Aphasia Re-Connect support groups at the Roberta Williams Speech and Language Therapy Clinic and Rowland Hill House Community Centre. Aphasia Re-Connect is a London-based charity that supports people with aphasia by providing a social community and group speech and language therapy. The three workshops took place approximately one week apart, with each lasting two and a half hours. All workshops were video and audio recorded to collect qualitative data from discussions. At the beginning of each session, participants seated themselves freely before the first author and an SLT initiated proceedings with an introduction and addressed any participant questions. Following this, participants were assigned to begin workshop activities at two large tables. The first and second authors then concurrently facilitated the activities, providing support and prepared materials at each table.

### Ethics statement and informed consent

Ethical approval for this research was granted by the King’s College London Health Faculties Research Ethics Subcommittee Project ID: LRS/DP-22/23-37286, on the 16/06/2023. All methods were performed in accordance with relevant institutional guidelines and regulations. Participants were recruited through an aphasia support charity, and research sessions were conducted at familiar community facilities to reduce travel-related burden. These locations were already known to participants through their regular support group sessions, and most participants attended independently via public transport. Informed consent was obtained from all participants prior to data collection, including consent for the publication of identifying information and/or images in this open-access publication. The authors affirm that human research participants (or their legal guardians) provided informed consent, for publication of identifying information in Tables 1 and 2. All participants were over the age of 18, and pseudonyms are used throughout to protect participant identities.

### Participants

Complete participant information is presented in Tables 1 and 2. In sum, 14 people with aphasia participated in the three workshops – 12 participants had moderate–severe aphasic language difficulties as a result of stroke, and 7 had right-side bodily paralysis from hemiplegia. Ages ranged from 35–73 years old with an average age of 60.8 years ($\sigma$ = 11.4). Furthermore 7 SLTs were employed as co-designers and to support the workshop activities. Only images of participants who consented to having their identifiable images published in articles and presentations are shown in this paper. The SLTs consent was gained by the first author via an approved consent form. Similarly, for the people with aphasia at the charity, consent was gained by an approved SLT via an accessible consent form and process. All participants were paid 20 GBP per hour. All names used are pseudonyms.

**Table 1:** Overview of 14 participants with aphasia across: co-design workshops. Assessed by an SLT are participants’ aphasic language – expression i.e., writing/speaking and comprehension i.e., reading/listening – scaled: Mild, Moderate and Severe; plus participants’ hemiplegia.

				Language			
Name	Gender	Age	Aphasia	Expression	Comprehension	Hemiplegia	Attendance
Steve	Male	52	Severe	Writing: Severe Speaking: Moderate	Reading: Severe Listening: Moderate	Yes	Workshop 1
Billy	Male	57	Moderate	Writing: Moderate Speaking: Moderate	Reading: Severe Listening: Mild	Yes	Workshop 1
Pete	Male	73	Moderate	Writing: Moderate Speaking: Moderate	Reading: Moderate Listening: Mild	Yes	Workshop 1; 2
Isaac	Male	70	Moderate	Writing: Moderate Speaking: Moderate	Reading: Mild Listening: Mild	No	Workshop 1; 2
Jess	Female	65	Moderate	Writing: Moderate Speaking: Severe	Speaking: Severe Listening: Moderate	No	Workshop 1; 2
Ella	Female	70	Severe	Writing: Severe Speaking: Severe	Reading: Severe Listening: Moderate	No	Workshop 2
Holly	Female	61	Severe	Writing: Severe Speaking: Moderate	Reading: Severe Listening: Mild	Yes	Workshop 2
Brian	Male	35	Severe	Writing: Moderate Speaking: Severe	Reading: Moderate Listening: Mild	Yes	Workshop 1; 3
Rowland	Male	51	Moderate	Writing: Severe Speaking: Moderate	Reading: Moderate Listening: Mild	No	Workshop 1; 2; 3
Gerald	Male	64	Mild	Writing: Mild Speaking: Mild	Reading: Mild Listening: Mild	Yes	Workshop 1; 2; 3
Ruby	Female	71	Mild	Writing: Mild Speaking: Mild	Reading: Mild Listening: Mild	No	Workshop 2; 3
Patrick	Male	59	Moderate	Writing: Severe Speaking: Moderate	Reading: Moderate Listening: Mild	Yes	Workshop 3
Joy	Female	51	Severe	Writing: Severe Speaking: Severe	Reading: Severe Listening: Moderate	Yes	Workshop 3
Edward	Male	51	Severe	Writing: Severe Speaking: Severe	Reading: Severe Listening: Severe	Yes	Workshop 3

**Table 2:** Overview of seven speech and language therapist (SLT) participants. Role refers to SLTs professional experience spent working with people with aphasia who supported participation.

Name	Gender	Role	Experience	Attendance
Bella	Female	SLT	1 year	Workshop 1; 2
Alice	Female	SLT	1 year	Workshop 1; 2
Carol	Female	SLT	1 year	Workshop 2
Tanja	Female	SLT	2 years	Workshop 3
Sophie	Female	SLT	40 years	Workshop 3
Samantha	Female	SLT	5 years	Workshop 3
Kate	Female	SLT	4 years	Workshop 3

### Workshop 1: exploring smartglass perspectives

Workshop 1 explored co-designers’ perspectives on diegetic smartglass prototypes featured in films and advertisements showcasing emerging mixed reality technologies. Video clips were deliberately selected to represent a diverse range of form factors, wearability, interaction types (public and private), and technological affordances. These clips were sourced from publicly available media, including both films and terrestrial advertising. Examples included fictional portrayals of smartglasses in popular films (e.g., Spider-Man: Far From Home, 2019), alongside recent promotional campaigns for translation-enabled and mixed reality headsets. All clips were used exclusively for the purposes of private research and workshop facilitation. For images of Workshop 1 refer to the supplementary materials (SM2).

To maintain engagement, the clips were relatively short, ranging from 31 to 88 seconds ($\bar{x}$ = 55.2, $\sigma$ = 15.3). While not exhaustive, we believe that using mass media clips provided an accessible way to elicit reflections on complex diegetic and MR technologies, including their form factors, user interactions, and affordances. The clips were introduced and played on a large screen for all co-designers. After watching each clip, co-designers participated in a tangible scenario grids activity designed to empower them to critique the video content and consider scenarios where the presented smartglass technology could be beneficial. To ensure accessibility, we employed a highly tangible discussion approach, incorporating a shared A1 page, sticky notes, and 36 tangible cards (each representing a video clip or discussed context). The scenario grids structured discussions by organizing insights into six columns: (1) the video clip, (2) opinions, (3) contexts of usage, (4) communication challenges, (5) people involved, and (6) potential affordances or solutions provided by the smartglass technology. Throughout the session, we encouraged co-designers to openly critique and envision practical applications of MR technologies.

### Workshop 2: low-fidelity smartglass prototyping

Workshop 2 focused on low-fidelity prototyping of smartglasses for video prompts, enabling co-designers to envision and prototype a range of MR technologies in an accessible manner. The video prompt scripts were derived from: (1) the scenario grids developed in Workshop 1, (2) extensive qualitative data from the research team’s previous studies with communities living with aphasia (e.g., ^24,26,29^), and (3) Parr’s research on social exclusion among people with aphasia^17^. Video footage was sourced from static images captured in prior research, open-source videos and images, GPT-4 text-to-image prompts, and AI-generated voice libraries. Each video depicted a fictional character with aphasia encountering personal accessibility challenges in a specific context. These scenarios were deliberately open-ended, avoiding constraints toward specific technologies to encourage divergent low-fidelity prototyping. To maximize accessibility, each video was paired with a tangible card summarizing the AI-generated fictitious character.

Following each video prompt, researchers facilitated 5-minute group discussions, answered questions, and used sticky notes to summarize the challenges faced by the fictional character. Co-designers then collaboratively envisioned and built MR smartglass technologies, with researchers overseeing fabrication based on established MR design guidelines^36^. Initially, lensless cardboard cutout glasses helped co-designers reflect on different smartglass form factors, aesthetics, and social acceptability. Additionally, craft materials (e.g., fabric, card, string) and constructor straws enabled co-designers to prototype MR assets, including their 3D size and spatial proportions. Some co-designers received assistance from SLTs or researchers for more dexterous building tasks. Others preferred working in small groups, with an SLT or researcher facilitating discussions. Throughout, researchers remained supportive and politely inquired about design choices. The workshop concluded with group demonstrations of co-designers’ smartglass prototypes. Images from Workshop 2 (SM3), three of the five video prompts (SM4) and tangible character cards (SM5) for introducing video prompt characters are available in the supplementary materials.

### Workshop 3: evaluating a HoloLens HMD smartglass application

In Workshop 3, people with aphasia freely evaluated and tested a smartglass HoloLens HMD application built by the research team to scaffold people with aphasia’s mobility and communication across a range of public contexts. The wearable application was based directly on findings from the first two workshops. Sequentially, participants engaged with accessible handouts providing an overview of device features, supported to calibrate the HoloLens HMD and freely test. One researcher/SLT supported participants’ full engagement with the high-fidelity smartglass prototype’s features. Following smartglass usage, participants completed a feedback questionnaire and held group discussions garnering consensus for future refinements. Images from Workshop 3 (SM6), a full demo video of the HoloLens HMD application (SM7) used in Workshop 3 can be found in the supplementary materials.

### Data analysis

All workshops were analysed for structured observation of the video data and transcribed using NVivo 14. The transcription included both verbal and non-verbal communication e.g., instances when co-designers with aphasia used pen/paper or bodily gesture. We then applied inductive Thematic Analysis – an iterative process whereby qualitative data is restructured into themes. In line with Braun and Clarke’s original interpretation^37^, the coding process was carried out by solely the first author, before all authors refined themes collaboratively. In total, we found 326 instances of discussion which were categorised into four themes. For workshop 2, the low-fidelity prototypes are categorised and discussed. Workshop 3 was video and audio recorded for data analysis. Whilst, quantitative data was compiled from the feedback questionnaires.

**Fig. 2:**
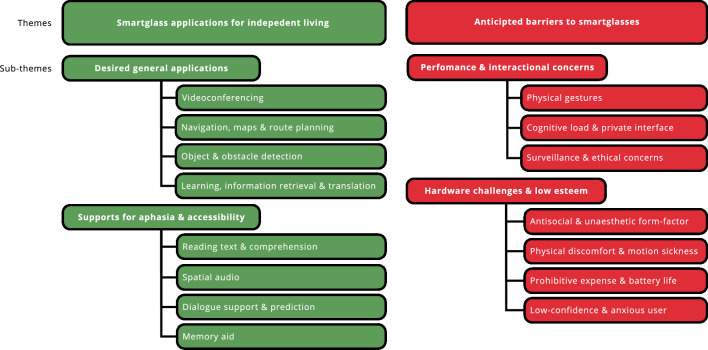
Results from thematic analysis comprising two themes and four sub-themes.

## Results

The qualitative perspectives of co-designers across all three workshops were thematically analyzed and compiled into a taxonomy with two main themes: (1) suggested smartglass applications for independent living and (2) anticipated barriers to smartglass adoption (see Fig. 2).

### Smartglass applications for independent living

Initially, co-designers with aphasia and SLTs envisaged **videoconferencing** via MR smartglasses. The option to resize MR videoconferencing screens was valued by co-designers, *“Bella: doing like a video call you could use this gesture [stretching arms] to pull the call and make it bigger... instead of an iPad you have a big thing you can manipulate”*. Leveraging smartglass videoconferencing for discreet support in public settings was recognised as helpful, *“Researcher 4: Imagine you could get your friend, [SLT] in as a hologram? Billy: At the moment there is nothing out there that helps!”*. Using smartglasses for accessible **navigation, maps, and route planning** was recognized as valuable by co-designers with aphasia. Initially, Gerald – a wheelchair user – envisaged leveraging MR’s 3D for step/elevation free access to different environments, *“Gerald: Cos! You look at a 2D-map and it doesn’t give hills, elevations and all of that [...] It highlighted a route that was stair free!”*. Compared to a smartphone, many believed that smartglasses would better support safe multitasking whilst walking,*“Pete: Be like maps! [...] Show you where you’re going [...] [Currently] people having the phone and getting run over! [They’re] Looking at the phone instead of looking where they’re going!”*. Building on this, many considered the potential of smartglasses to support **object and obstacle detection**. For instance, co-designers with mobility challenges wanted peripheral recognition of obstacles during walking, *“Gerald: Yes and say ‘stop!’ [...] I’m constantly tripping over things in my flat! [...] Shoes! God yeah!”*. Others desired object detection to assist with finding misplaced belongings at home and shopping items in the supermarket. Finally, co-designers considered leveraging smartglasses for proactive **learning and information retrieval**. Some co-designers acknowledged it would be helpful to have projected/floating MR cooking food recipes/calories. Plus, for analysing extensive search engine results, *“Rowland: Instead of putting a list of restaurants up on your hand! Projected on the wall!”*.

Regarding aphasia-specific applications, many desired audio-driven smartglass interactions for **reading text and comprehension**, *“Researcher 1: So audio would be easier? Steve: If you looked and it says mug rather than writing mug”*. Building on this, some envisaged MR smartglass visuals to improve understanding and comprehension during conversations with medical professionals, *“Gerald: A 3D image of a torso and the doctor could go where [is the pain]? [...] Bella: As a diagnostic aid!”*. Next, co-designers envisioned smartglass **spatial audio** in noisy locations, *“Gerald: When you run into a group you get too many people speaking at once! Bella: So yeah if the camera had the auditory input of whomever you are looking at!”*. Plus, for recognition of contextual objects, *“Researcher 1: So would you like it played to you in your ear? Steve: Road, Road, Road... you see!”*. Following this, co-designers discussed MR assets designed to enhance their communication by either **dialogue support and prediction**. Particularly, during stressful communication episodes when word recall is challenging, *“Steve: Going McDonald’s – I can’t spit the words that I want! And have it [smartglasses] talk out [gesturing with hands outwards]!”*. Lastly, co-designers felt MR assets could be used as a **memory support** within smartglasses. For instance, MR assets aiding memory retrieval and storytelling, *“Pete: I think it would be useful! If you could bring the images! Researcher 4: So you can show me!”* and *“Billy: Yeah! Happy memories are good memories!”*. Other functional memory supports from co-designers included, MR projected personal information to support recollection when filling out complex forms (e.g., hospital/benefits) or as an SLT noted, *“Alice: I’d like them during exams!”*.

### Anticipated barriers to smartglasses

Multiple co-designers expressed concern with smartglasses interaction accessibility for people living with aphasia. For many, smartglasses dependence on predominantly **physical gestures** was deemed inaccessible – particularly for people living with bodily paralysis onset by stroke. In response to two-hand smartglass MR gestures, a co-designer with hemiplegia noted, *“Brian: I have one hand! And this is no hand! Researcher 4: You can’t do the two hand gesture? Brian: Yeah!”*. Furthermore, some of the *more* fine gestures (e.g., pinching) were deemed a barrier, *“Alice: There is an accessibility issue isn’t there! For some people trying to manipulate something small! Doing that [swipe with arm] is a lot easier”*. Co-designers expressed concern with smartglasses visual clutter, **cognitive load and private interface**. In response to multiple smartglass displays, *“Pete: It’s distracting! Be distracting! [..] Brian: it could be distracting as well! Pete: Too much to process! You put glasses on like that – you’d go mad!”*. As a consequence, many desired intuitive control, *“Isaac: You’d have to have the ability to stop it very quickly!”* and prevent smartglasses from becoming, *“Gerald: A bit bloody annoying after a while”*. Many also acknowledged that the complex, private interface of smartglasses would make it more challenging to seek repair or assistance from others compared to smartphones. Some co-designers acknowledged **surveillance and ethical worries** with smartglasses. Many expressed discomfort with smartglasses recording your gaze history via cameras/GPS, *“Rowland: Will it, will it, will, would it keep your history? [...] Bella: How long is the recall on it? Rowland: No, I wouldn’t want it then!”*. Regarding smartglass cameras photographing memories – many envisaged it could be a *‘slippery slope’* and lead to surveillance. Elsewhere, many expressed hesitancy with providing intimate bio-data to devices including electroencephalogram (EEG)/retinal scans, *“Gerald: What if it was retrieving something from inside your brain?”*.

The most prominent critique centred on smartglasses having an **antisocial and unaesthetic form factor**. Many complained about the Vision Pro HMD being too socially prominent, *“Gerald: They’d have to make it like a pair of glasses. Bella: They are absolutely massive!”*. Consequently, many feared social judgement if they publicly wore socially prominent smartglasses – *“Pete: [The Vision Pro] its not normal glasses! They look like diving glasses! They look really silly! Researcher 4: They look silly? Pete: Yeah! Researcher 4: if your family came home and they saw you wearing those...? Pete: They’d lose the plot! Isaac: Yeah! Pete: They’d... think you would have gone mad!”*. Many noted that smartglasses could inhibit mutual eye contact – a key form of non-verbal communication – *“Pete: If it was normal glasses – but having them diving glasses on is silly! Isaac: Yeah! But you wouldn’t know what he was looking at! Brian: Yeah! Isaac: Whether he was looking at you or not! [...] Complete rubbish! Imagine walking around with a pair of them on!”*. Also, the prominent form-factor raised concerns that smartglasses could cause **physical discomfort and motion sickness**. Concerning motion sickness, *“Researcher 4: Yeah! Would smartglasses make you feel sick? Pete: A bit! Isaac: Well yeah! It didn’t me! But I can understand people who would errr... get sick!”*. Co-designers expressed disappointment with smartglasses **prohibitive expense and battery life**, *“Researcher 1: The [Vision Pro] cost almost $4000. Steve: [Ironically] That’s cheap! Rowland: Come on man... that’s ridiculous! [...] They could get err... robbed!”*. Battery life was also noted, with many expecting more than 2.5 hours from a Vision Pro HMD – *“Rowland: What’s the battery life? Researcher 1: It could last about a day? Rowland: Ah fuck no!”*. Multiple co-designers acknowledged that they were a **low confidence and anxious user** of technology. Co-designers expressed concern that smartglasses could perpetuate frustrations and unwanted interdependencies, *“Ruby: But something like that! If his intellect is intact and he program it! [...] he relies on someone else to program it!?”*. Even noting that smartglasses were for a younger user-base, *“And are their skills in tech enough to cope with all the things your suggesting! [...] but an older person... And for myself – I would struggle to work with all that technology!”*.

### Low-fidelity smartglass prototypes

In response to video prompts we recorded co-designers 10 low-fidelity smartglass designs – these have been categorised into: (1) form-factors, (2) visual media and (3) audio interactions. Please refer to the supplementary materials (SM8) for categorised images of co-designers’ low-fidelity smartglass prototypes.

**Smartglass designs represent personal style.** Co-designers expressed differing personal preferences for smartglass design, even creating three ‘ideal’ personal form factors. Throughout, co-designers fiercely debated frame/lens designs for different faces, *“Bella: Depends on the face shape! Researcher 1: Yeah that’s true! Bella: If you’ve got a small face – oversized looks crazy! If you’ve got a little face and these massive glasses – it’ll look like you’ve got your mum’s glasses on! Rowland: Hahah!”*. Many co-designers owned multiple pairs of glasses and preferred different designs/shapes depending on context, *“Rowland: You’d be surprised errr... - I’ve had so many glasses! [...] When I look at them oh jheez – I wasn’t wearing that! Bella: [...] all the big, massive 80s glasses are popular again!”*. Some admitted to indecisiveness and strong emotions on glasswear, *“Rowland: Yeah! I got those! NHS glasses! I hated them when I was younger! When I was in school – I used to get people to sit on my glasses – I just wanted them broken!”*.

**Smartglass navigation and maps.** Initially, co-designers suggested arrow-based navigation/directives within the smartglasses, *“Rowland: You could have! Errr... No you could have arrows!”* and *“Bella: Like a little arrow”*. Beyond this, co-designers proposed maps for ensuring successful navigation. Particularly for finding grocery items 3D-maps were posited as helpful, *“Pete: Maps for that store [...] Gerald: Then I say if it was a 3D map it would highlight where beans are!”*. Co-designers employed constructor straws to determine readable distances for 3D visual assets, *“Bella: You need to look like it’s a bit further away – because else you can’t see it cause it’s too close!”*. To minimise clutter/distraction, co-designers suggested that arrows should not be central – instead favouring cues on the periphery of the wearer’s visual field i.e., *“Isaac: Top left!”*. Equally, co-designers emphasised users differing *“Gerald: Focal point!?”* preferences.

**Smartglass speaking notes, videos and keywords.** Speaking notes provided by smartglasses were considered useful for prompting during pressurising dialogues. Co-designers even co-designed smartglasses prompts for ordering in busy cafes, *“Pete: discreet smartglasses! Might help! [...] Pete: Bring up what you want to say on the screen! Researcher 4: So you might have some phrases that he’s written down before – just to remind him!”*. In contrast, Pete suggested video-based support for prompting communication during ordering, *“Pete: If his wife sends him a video of what she wants – saying what she wants!”*. To ensure a readable interface design co-designers highlighted the importance of suitable size/contrast, *“Gerald: Also you have to make sure that it’s a contrast that John is able to read! Isaac: It would have to be bigger than that! [...] Pete: That’s a better size – yeah! Researcher 4: How does that look? Pete: That’s fine! Researcher 4: That sort of size is good? Pete: Depends what the background is like but yeah! Gerald: Like I say it’s got to be contrasted with the background! Pete: Nice black background and the letters are in white!”*.

**Private and public smartglass audio feeds.** For co-designers with aphasia, private/public audio was deemed a greatly beneficial smartglass modality. Co-designers made smartglasses for personal in-ear audio cues, *“Rowland: Just like predictive texting!”* and *“Carol: They could connect to like little earphones that could speak to you!”*. Co-designers recognised that navigation could be deduced from smartglass audio feed, *“Rowland: Talking in each ear for left and right!”*. Plus, for support with word-finding, *“Rowland: It will come up with three different words and you point at it and it goes [generates audio for] that one!”* and to *“Rowland: Help you say you need time to talk”*. Co-designers designed smartglasses that publicly output loud audio if non-verbal, *“Steve: And have it [smartglasses] talk out! [gesturing hands]”*. Albeit the smartglasses *must* be loud enough for busy contexts, *“Gerald: the smart glasses if there is an external speaker! Researcher 1: Okay! Pete: Would it be loud enough though? In terms of the speakers – they’re so quiet! With the coffee shop being so crowded!”*.

**Fig. 3:**
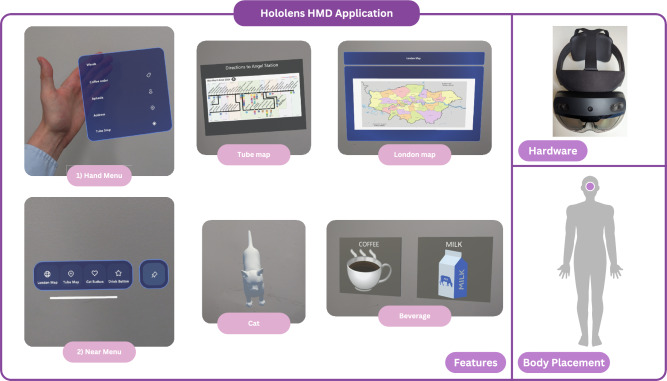
High-fidelity application developed for Workshop 3 using HoloLens HMD. Series of images of applications key features including Hand Menu of 4 audio-based language prompts and Near Menu of 4 head-tracked private language assets.

### Evaluation of a HoloLens HMD smartglass application

For Workshop 3, we designed and built a high-fidelity smartglass application to scaffold and support people with aphasia’s communication, mobility and navigation across a multitude of public locations. Pictured in Fig. 3, the features of the smartglass application were derived from co-designers’ shared perspectives (see Fig. 2) and low-fidelity prototypes – the smartglass speaking notes, maps and audio feeds. Concerning smartglass hardware we used a HoloLens 2 HMD. In terms of software, the wearer is provided with 2 menus of smartglass assets. Initially, the *Hand Menu* presents 4 audio-based language prompt buttons – these audio assets can be synthesized both privately/publicly thereby either supplementing or replacing speech. In the current version, the four synthesized messages are: (1) *“I would like a black coffee with three sugars”*, (2) *“I am trying to get to Angel Station”*, (3) *“I am heading to Aphasia Re-Connect”* and (4) *“I have had a stroke and aphasia”*. Meanwhile, the head-tracked *Near Menu* provides 4 head-tracked private assets. Firstly, a button provides a map of the transport network. Secondly, a button provides a re-sizable/movable map of London boroughs. Thirdly, a button for a 3D cat. Finally, a button triggering symbols of coffee/milk to facilitate Wizard-of-Oz testing of smartglass symbol-based speaking notes. All menus support multimodal input – buttons are pressable using hand gesture, eye-gaze or keyword utterances. The software was developed in Unity 2022.3 LTS, Visual Studio 2022 and written in C# and C++. Development employed the Windows 10 SDK, Mixed Reality Toolkits (MRTK 3), Open XR plugin and Wikimedia image assets.

#### Quantitative results

**Fig. 4:**
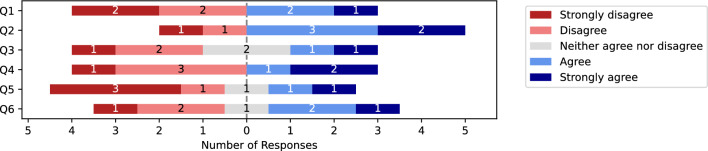
Recorded Likert responses to HoloLens HMD smartglass application evaluated during Workshop 3. Participants responded to the following questions: (Q1) The Hololens was easy to use. (Q2) I enjoyed using the Hololens. (Q3) I would use the Hololens to support my communication. (Q4) It helped that the Near Menu could project maps, cat and beverage options. (Q5) The Hololens could support my communication in public. (Q6) The Hololens Hand Menu of audio prompts could support my communication.

Presented in Fig. 4, 42 Likert ratings were recorded. From this, there was 8 instances of ‘strong agreement’ for positively phrased questions. Next, there was 10 instances of ‘agreement’. Whilst, there was 4 instances of ‘neutrality’. In contrast, there was 11 instances of ‘disagreement’. Finally, there were 9 instances of ‘strong disagreement’. Collectively, the feedback was mixed with 18 ratings for positively phrased questions versus 20 ratings for negatively phrased questions In particular, the HoloLens received 5 instances of ‘strong disagreement’ in relation to: the potential of the device to support communication in public (N=3) and ease of use (N=2).

#### Qualitative findings

Most co-designers had limited experience with smartglasses and reacted excitably, *“Patrick: It’s Amazing! [...] Oh my god you look fantastic!”*, *“Joy: Oh my god! [...] Wow this is good!”*, *“Tanja: Very fun and different”* but would take time *“Rowland: getting used to it!”*. During testing, the smartglasses were successfully eye-calibrated for all participants, *“Ruby: You could imagine in an old people’s home – everyone using one of these”*. A hemiplegic user, Brian could successfully activate buttons and menus via simultaneous eye-gaze, one-hand gestures and verbal utterance of keywords, *“Brian: Only can do one hand!”*. Participants could use the HoloLens smartglasses whilst wearing prescription glasses and desired a version that *“Gerald: Would be good with [built in] prescription lenses! [...] Very fun!”*. Specifically, Tanja and Samantha both felt the, *“Samantha: Hand Menu was most useful for spoken prompts”* and suggested further options. In response to the MR maps, Sophie was enthused *“Sophie: It’s quite useful [...] It’s a nice clear tube map – I’ll give you that!”* and quickly usable. Elsewhere, Patrick and Samantha both liked the beverage options and felt it would be helpful for ordering, *“Patrick: Cappuccinos, Lattes and other drinks”* in a café or providing, *“Samantha: Contextual subtitles for people who find reading helpful but don’t want the AAC to speak for them”*. Ultimately Patrick appreciated HoloLens was a *“fascinating concept full of bugs – but I’ve never seen anything like it before!”*.

Collectively, participants believed the HoloLens smartglasses were still a work-in-progress. Predominantly, control by physical gesturing was challenging for people living with post-stroke aphasia, *“Rowland: Work in progress! It could be very confusing if you just awoke from stroke [..] not used to touching stuff”* and *“Gerald: I think it might get better in a few generation’s time!”*. Similarly, participants had difficulty using menu buttons/moving MR assets: *“Brian: Jesus Christ [trying to get London Map asset out of the way] Come on! Ah! [pinch] Ah fucking hell! I want it to go away!”* and *“Rowland: That’s hard [tapping play button]... it’s like it’s floating! This is kind of mad! I can’t hit the play! [...] Weird trying to push things!”* – ultimately limited tangible feedback from MR buttons was prohibitively challenging. Another participant, Joy, living with hemiplegia and a braced left-arm struggled with all multimodal interaction (i.e., pressing buttons, uttering keywords and eye-gaze) and could not easily interact with menus causing frustration, *“Joy: But I can’t!! [...] I can’t do it”*. After multiple attempts, the lack of control at times caused overwhelm, *“Rowland: This is too overwhelming... err... for a person who had a stroke err... it would be hard to control! It would make you frustrated!”*. Equally, Edward also faced difficulty with the headset recognition of hand gestures – yet successfully resized maps with arm gestures and appreciated the feeling of being present in the room, versus VR.

Many noted potential embarrassment from gesturing and interacting with the HoloLens smartglasses in public, *“Gerald: I must admit... it looks weird! [In response to gestures]. That looks even more weird! He’s interacting with something that is not there!”* albeit enjoyable, *“Gerald: it’s huge enjoyment for the people watching!”*. Consequently, Joy suggested that smartglasses would be more useful for gaming at home as it looked, *“too strange”* for usage in public. Notably, Patrick and Sophie argued that *Holo AAC* was too private and could hinder shared communication, *“Patrick: You don’t have the same view – even if it was on an LCD!”* and *“Sophie: [You could] lose shared experience with others”*. Ultimately, the present cost of the HMD was unjustifiable, *“Rowland: So what is the cost again? Researcher 1: About $4000! Rowland: Good god! Brian: Wow!”*. The pointers extruding from fingers within the HMD left many confused, *“Tanja: They threw me about pressing [...] Until, once you get used to putting your finger on it!”*. Patrick who lives with hemiplegia could quickly select the smartglasses Near Menus with a one-hand gesture; yet he found the two-hand gesture of the Hand Menu challenging, *“Patrick: As I can’t use my other hand... but I can use a computer really, really well!”*.

## Discussion

The release of the Vision Pro^38^ and the public demo of the Orion smartglasses^39^ mark a new era for advanced consumer computing technologies and signal growing mainstream interest in smartglasses. Concurrently, advancements in large language models, multimodal AI interaction, smart assistants, and intelligent agents could further accelerate smartglass adoption by improving their functionality, assistance, and utility for communities living with disabilities^40^. In this paper, we present emergent insights into the potential of co-designing smartglasses to support people living with aphasia in London. Throughout the process, co-designers expressed strong enthusiasm for the potential of smartglasses to assist their daily lives. Consequently, continued research is essential to ensure these emerging wearable technologies support the independence and well-being of underrepresented communities, including those with complex communication needs due to aphasia and mobility challenges from hemiplegia. The PAOLI framework proposed by Charalambous et al.^41^ could serve as a valuable reference for future co-creative technology development with people with aphasia.

In early co-design sessions, participants with aphasia envisioned a range of immersive smartglass applications to support their independence, mobility, and communication. Proposed applications included videoconferencing, navigation guidance, obstacle detection, and environmentally adaptive information retrieval for public navigation (e.g., stair-free access routes). Notably, these supports should be contextually timed to assist during stressful mobility and communication episodes. Towards this goal, machine learning algorithms developed by Kong et al.^42^ have demonstrated how smartglass cameras can enhance contextual interaction, highlighting their potential for adaptive assistance. Additionally, our study identified that multimodal supports – such as text comprehension aids, dialogue prediction, spatial audio interactions, and memory assistance – could significantly improve public communication for people with aphasia. Notably, we found that smartglass interactions in public settings should prioritize discretion. Lee et al.’s^43^ systematic review recommends leveraging external devices (e.g., smart rings) and hands-free interactions (e.g., gaze tracking) to maintain user privacy and ensure seamless public support for wearers with disabilities.

Co-designers then created a range of tangible, low-fidelity prototypes, reinforcing prior research that tangible co-design with people with aphasia is a vital method for fostering mutual understanding^12^. Among the prototypes, arrow-based directives and 3D maps emerged as effective navigation aids, with peripheral versions deemed essential to avoid interfering with the wearer’s general mobility and walking. Building on this, co-designers recognized that speaking notes would need appropriate sizing and contrast to remain visible across different public settings. Additionally, they developed both private and public audio-based interactions to support dialogue in high-pressure situations. They emphasized that smartglass speakers must dynamically adjust volume to accommodate varying background noise levels, ensuring usability across diverse, noisy environments such as public transport, shops, and restaurants. Notably, research by Ahmetovic et al.^44^ has focused on optimizing screen reader intelligibility in noisy settings for BLV users – similar optimizations will also be essential for ensuring smartglass accessibility.

A key finding from this research is the importance of smartglasses offering a stylish means of support for people with access needs. Co-designers prioritized eyewear designs that reflected their personal style, favoring bold aesthetic choices such as colorful and prominent smartglass frames. Traditionally, assistive technology for older adults and people with disabilities has prioritized functionality and public discretion^7^. However, designer Graham Pullin^7^ argues that such neutral designs reflect an implicit sense of shame regarding the assistive technology. In contrast, our co-designers’ preference for vibrant and noticeable designs challenged these conventions. This research also identified anticipated barriers to smartglass adoption among people living with aphasia. Noted concerns included hesitancy and fears regarding smartglass surveillance – particularly relating to the multiple cameras embedded in the smartglasses. To address this problem, some recent smartglasses have incorporated visible green-light indicators to signal when their cameras are active^45^. However, notable research from Opaschi et al.^46^ has identified the numerous security and privacy threats that materialise through public interactions with smartglasses equipped with embedded cameras. Beyond smartglass accessibility, future development must continue to prioritize user privacy and security – including transparent data capture practices, consent mechanisms and safeguards to preserve users safety and autonomy.

While the HoloLens prototype served as an effective high-fidelity proof of concept prototype, we acknowledge that the current hardware is prohibitively bulky and expensive for widespread use. Future research should explore how the recommended accessibility features could be adapted to support navigation and communication for people with aphasia on increasingly lightweight and affordable commercial smartglasses and other emerging wearable devices^47–49^. Future collaborations with industry partners will also be essential to refine these prototypes for real-world use and ensure that accessibility innovations can scale into consumer-facing products. Continued co-design with diverse populations and iterative development will also be key to progress towards functional and inclusive smartglass technologies. Finally, the high cost and limited battery life of smartglasses were recognised as significant deterrents, underscoring the need for these devices to be both affordable and capable of lasting a full day of typical use. In response to battery constraints, the Orion smartglasses pair with a compact Bluetooth puck for off-device computing, helping extend battery life^39^.

Many of the smartglass functionality barriers anticipated by co-designers with aphasia became evident during our testing of HoloLens HMDs. Co-designers found the bulky hardware impractical for public use and were unimpressed with its overall design. Additionally, the SDK’s touchless free-hand gestures – required to operate the HoloLens – were largely inaccessible, particularly for co-designers with post-stroke hemiplegia and bodily paralysis. Movements such as pinching, navigating menus, and pressing virtual buttons without tactile feedback proved especially difficult. These complex touchless interactions created significant cognitive load, with many co-designers finding the management of multiple HMD menus overwhelming and exhausting. In contrast, most VR devices still incorporate external remote controls for improved interaction^43^, and Xbox’s Adaptive Controller creates custom interaction experiences to ensure gaming accessibility^50^. We recommend future smartglass research continue to explore tangible external devices and controllers as alternative interaction methods to enhance accessibility. While our study has considered early-stage co-design and proof-of-concept evaluation, the long-term feasibility of implementing smartglasses as a scalable assistive technology will depend on several key infrastructural factors. These include funding mechanisms to ensure equitable access, training programs for both end users and clinicians, and the provision of technical support and repair pathways^51^. Without established infrastructure, even the most accessible smartglass designs risk becoming underused or abandoned in practice^52^. For communities living with aphasia, existing research has focused on community ecosystems to support long-term use of AAC devices^27^, provision of mental health services^53^, and language rehabilitation^54^. Similarly, future work will have to consider the broader socio-technical systems required to support sustained smartglass adoption.

Concerning study limitations, all participants were recruited through a single UK-based aphasia charity, which may limit the cultural, linguistic, and socioeconomic diversity of perspectives captured. As such, findings may not fully generalize to the broader population of people with aphasia, particularly those underrepresented in digital or healthcare inclusion efforts. Future work should actively seek to engage more diverse communities.

## Conclusion

In this work, we co-design smartglass technologies with people living with aphasia. To start, we began critiquing media from film and advertising of the latest smartglass technologies. The presentation of these technologies supported collaborative envisioning and served as a springboard for co-designers low-fidelity prototyping of different smartglass form-factors and applications. Outputs from this enabled us to build and test a high-fidelity HoloLens smartglass prototype and application to test functionality. Consequent insights from this work can inform: the development of accessible smartglasses, co-design research and promotes more aesthetic assistive technologies.

## Supplementary Information

Supplementary Information 1.

## Data Availability

To protect participant anonymity, full workshop transcripts are not publicly available. However, all qualitative data supporting the findings of this study are included within the paper. Additional anonymized data may be available from the corresponding authors upon reasonable request and subject to ethical approval and participant consent, where applicable. The following supporting materials are provided in the supplementary files: SM1: GRIPP2 Short Form checklist, used to report patient and public involvement in line with best practices in participatory research. SM2: Three images of participants engaging with scenario grid activities during Workshop 1. SM3: Series of images from Workshop 2, co-designers using various materials – such as plastic glass frames, paper, pens, scissors and constructor straws – to prototype smartglasses and MR assets. SM4: Three of the five adapted video prompts shown to participants during Workshop 2 for ideating low-fidelity AAC prototypes. SM5: Tangible and accessible character cards used to introduce video prompt characters in Workshop 2. SM6: Series of images showing co-designers testing and evaluating the high-fidelity HoloLens HMD in Workshop 3. SM7: Demonstration video of the HoloLens MR headset application developed, evaluated, and tested by participants in Workshop 3. SM8: Images of low-fidelity smartglass prototypes built by co-designers in Workshop 2 categorized into three envisioned aspects: (1) form factors, (2) visual media, and (3) audio interactions.
