# Supplementary material for: Making smartglasses accessible: perspectives and prototypes from co-design with people with aphasia
Source: Sci Rep. 2025 Nov 3;15:38309. doi: 10.1038/s41598-025-22253-2 (PMC12583751; doi:10.1038/s41598-025-22253-2)
Supplement: Supplementary file 1 — Supplementary Information 1. [file 41598_2025_22253_MOESM1_ESM.zip › Supplementary/SM6.pdf]

(1) Co-designers with hemiplegia tests headset Near Menu

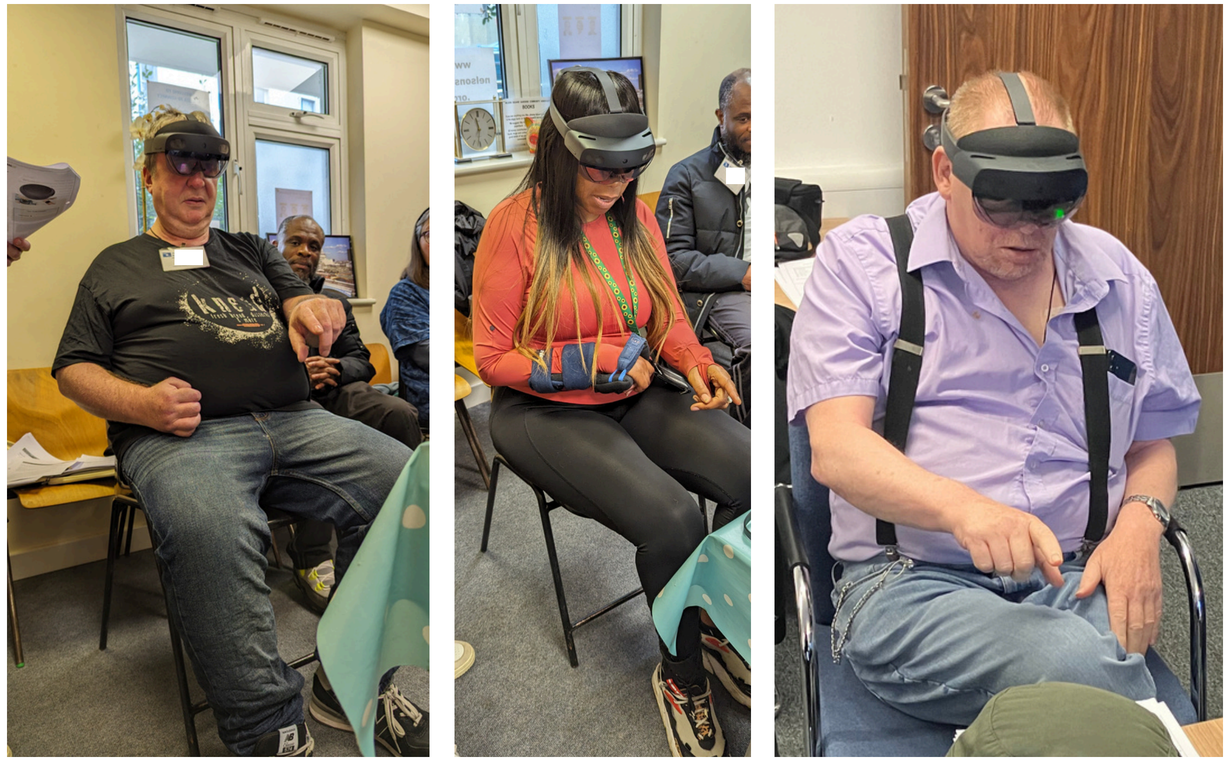

(2) Testing headset Hand Menu

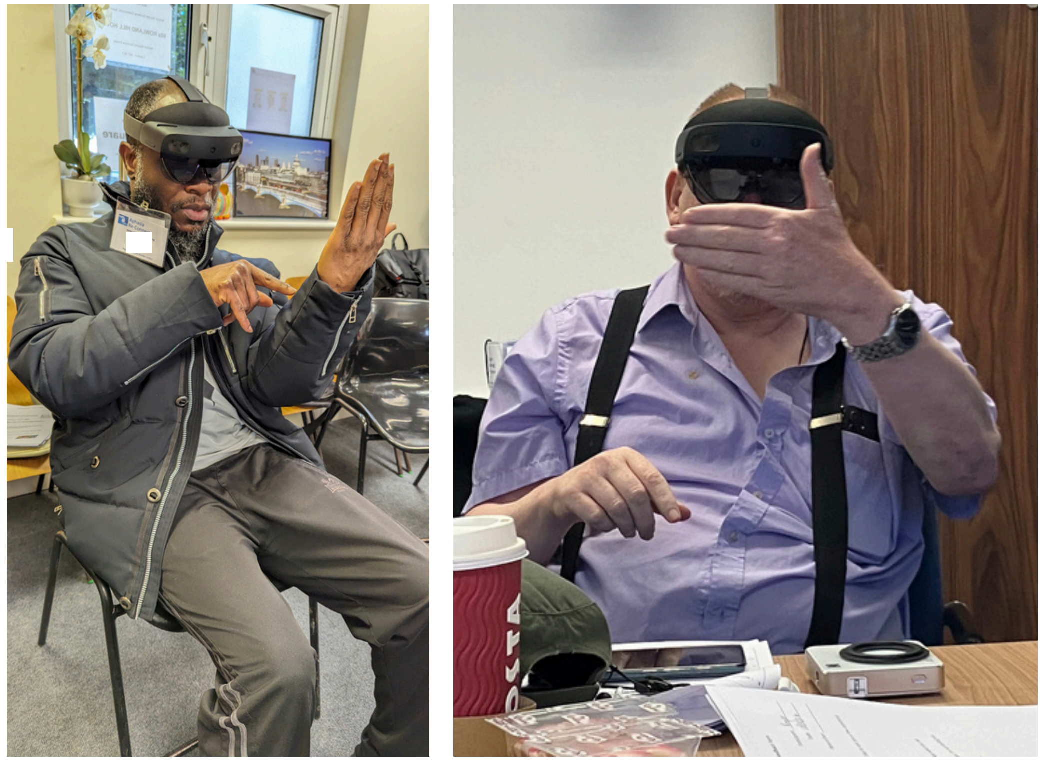

(3) Co-designers assess discreetness of headset gestures

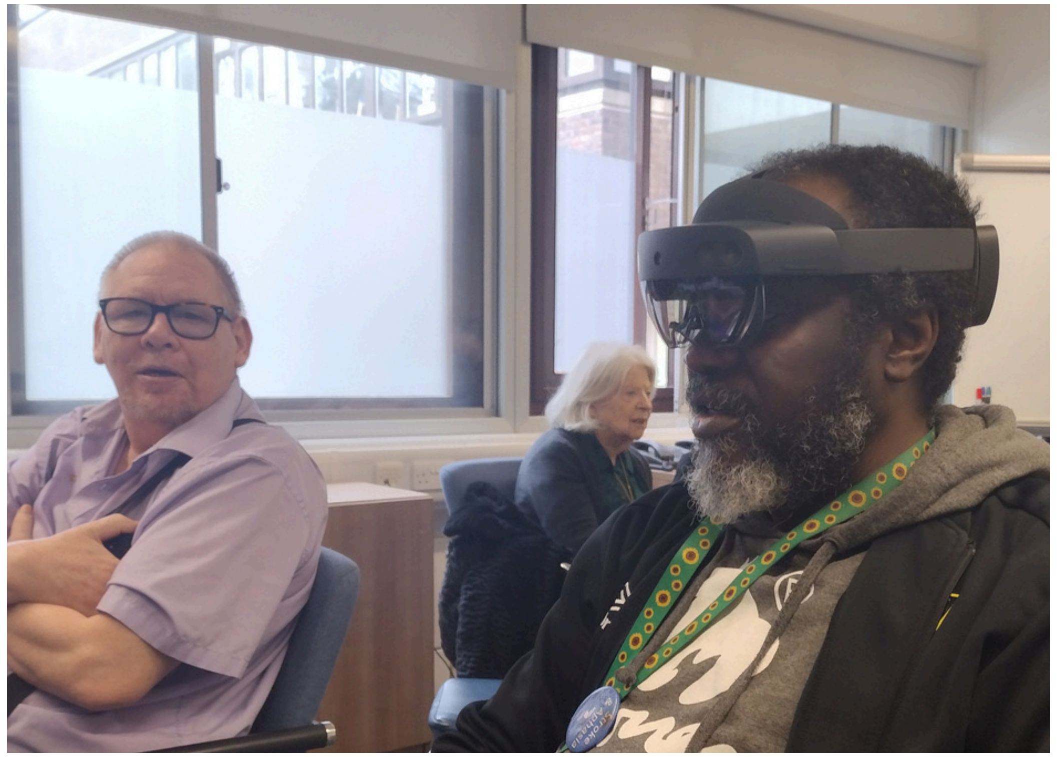

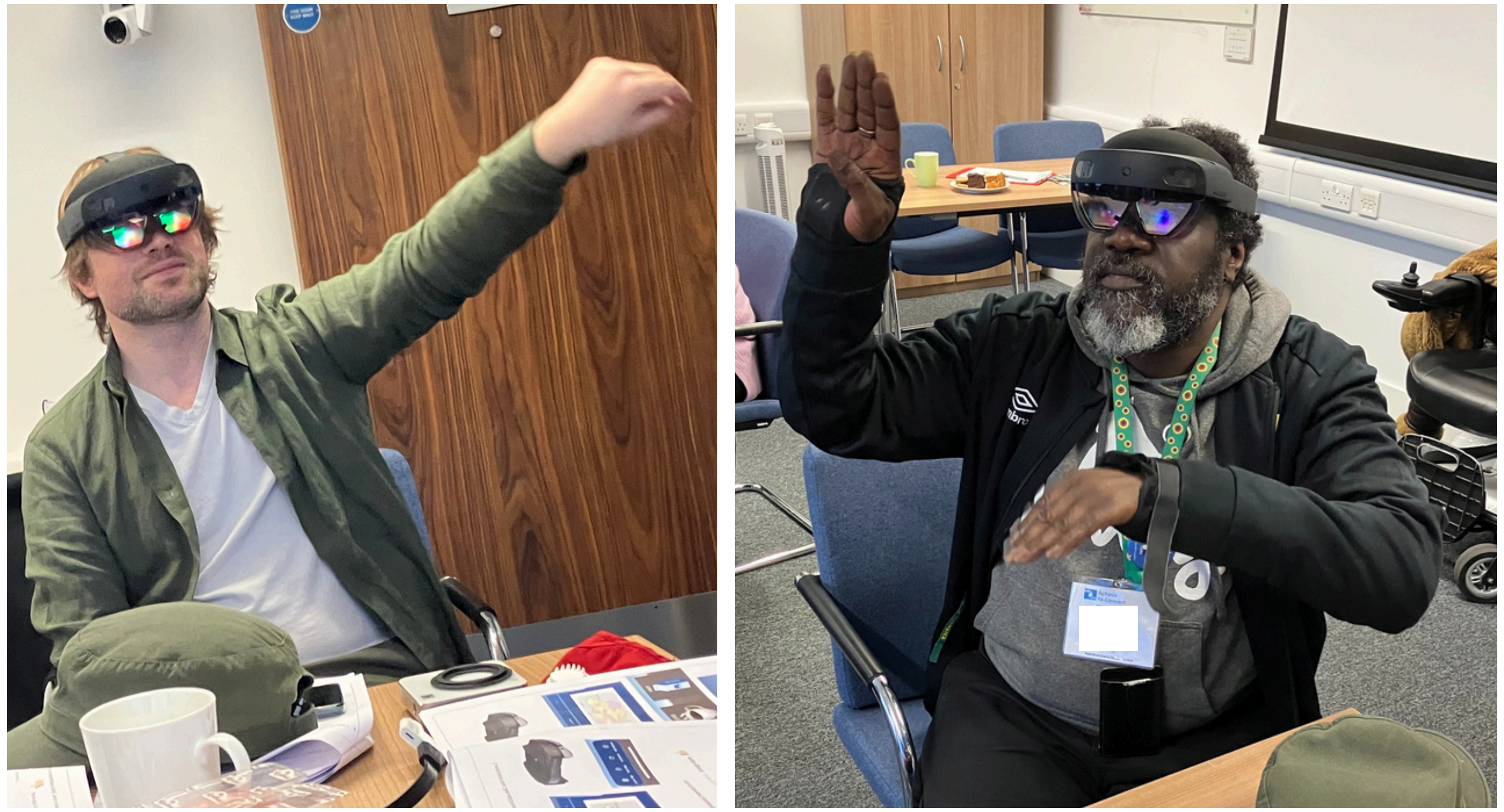

(4) Co-designers with hemiplegia tests scalable London Maps

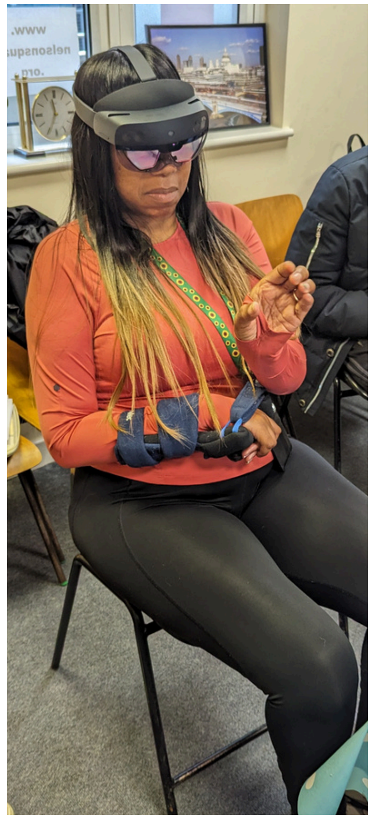

(5) Pinching & adjusting assets

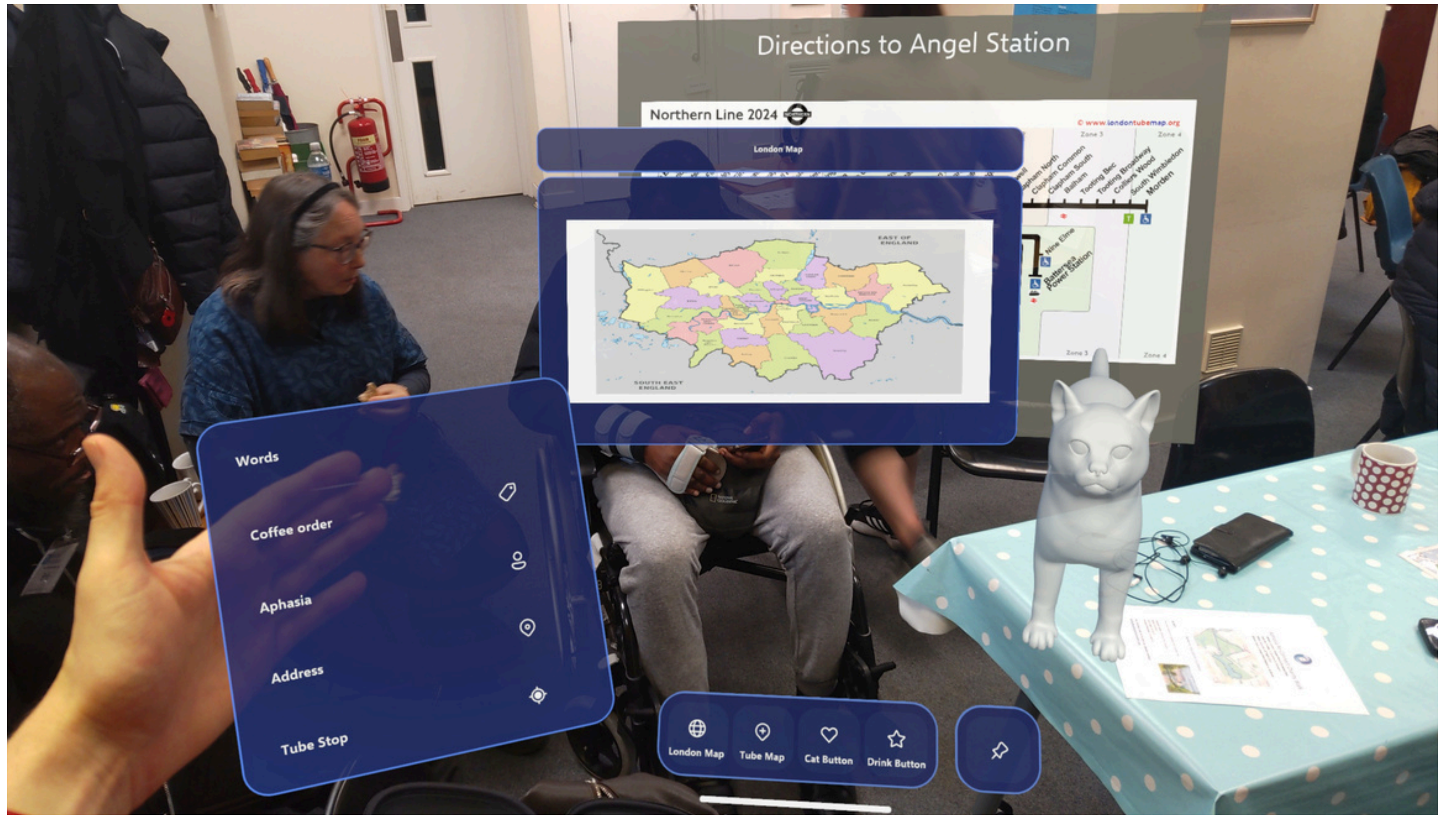

(6) Headset beverage symbols, Hand Menu, Tube Map, London Map & Near Menu deployed
