# Supplementary material for: Making smartglasses accessible: perspectives and prototypes from co-design with people with aphasia
Source: Sci Rep. 2025 Nov 3;15:38309. doi: 10.1038/s41598-025-22253-2 (PMC12583751; doi:10.1038/s41598-025-22253-2)

JOHN

- John is **80 years old**
- He lives with his **wife** in North London
- John is very **determined** and does not let his aphasia define him.
- He is an active **hobbyist** enjoying board games and film.
- Since his stroke, he has lived with aphasia for **12 years** – he is generally **partially verbal** and uses a walking stick due to **hemiplegia**

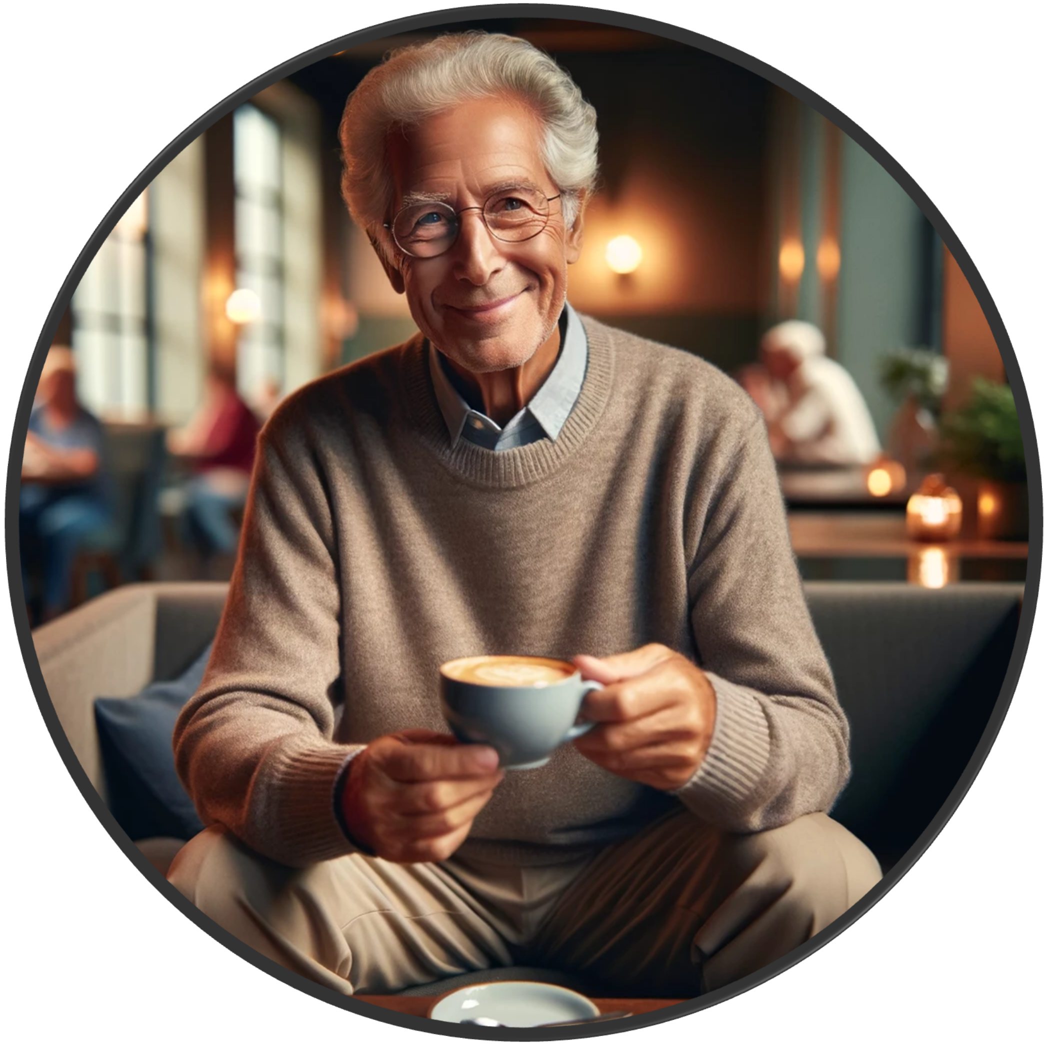

AMY

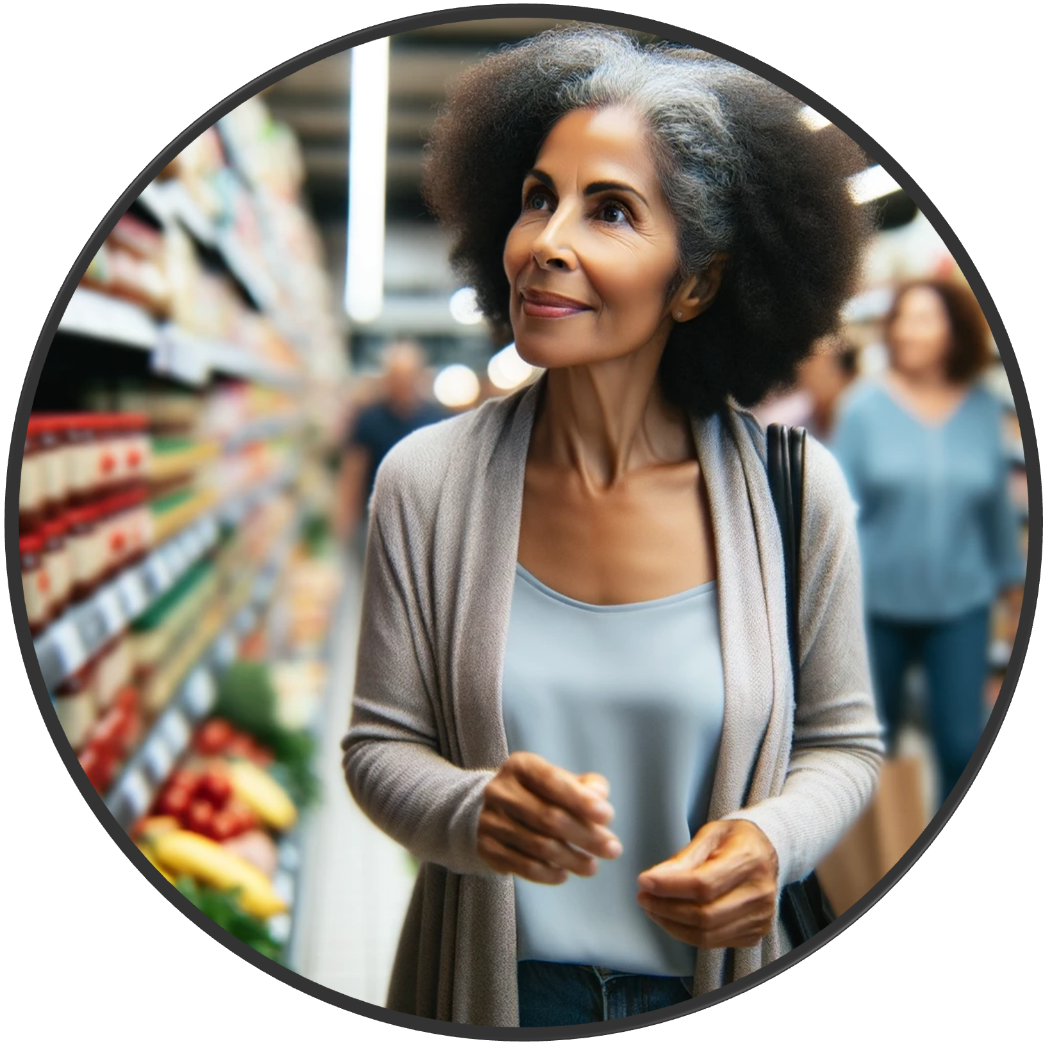

- Amy is **54 years old**
- She has a keen **sense of style** and loves travel. Amy is a proud **mum** of two boys and lives in East London.
- She has had aphasia and **vision difficulties** for 2 years since her stroke
- Amy used a computer in all her previous jobs and currently uses her smartphone effectively through **text to speech** as she cannot read

SARAH

- Sarah is **70 years old**
- She lives in Southwest London. Sarah is **highly knowledgeable** about all things art and undertook a history of art degree throughout the pandemic
- Sarah is an **active adopter of technology** and was even taught to post tweets about art by her grandsons in the summer
- She has lived with aphasia for 5 years since her stroke and uses a **wheelchair**

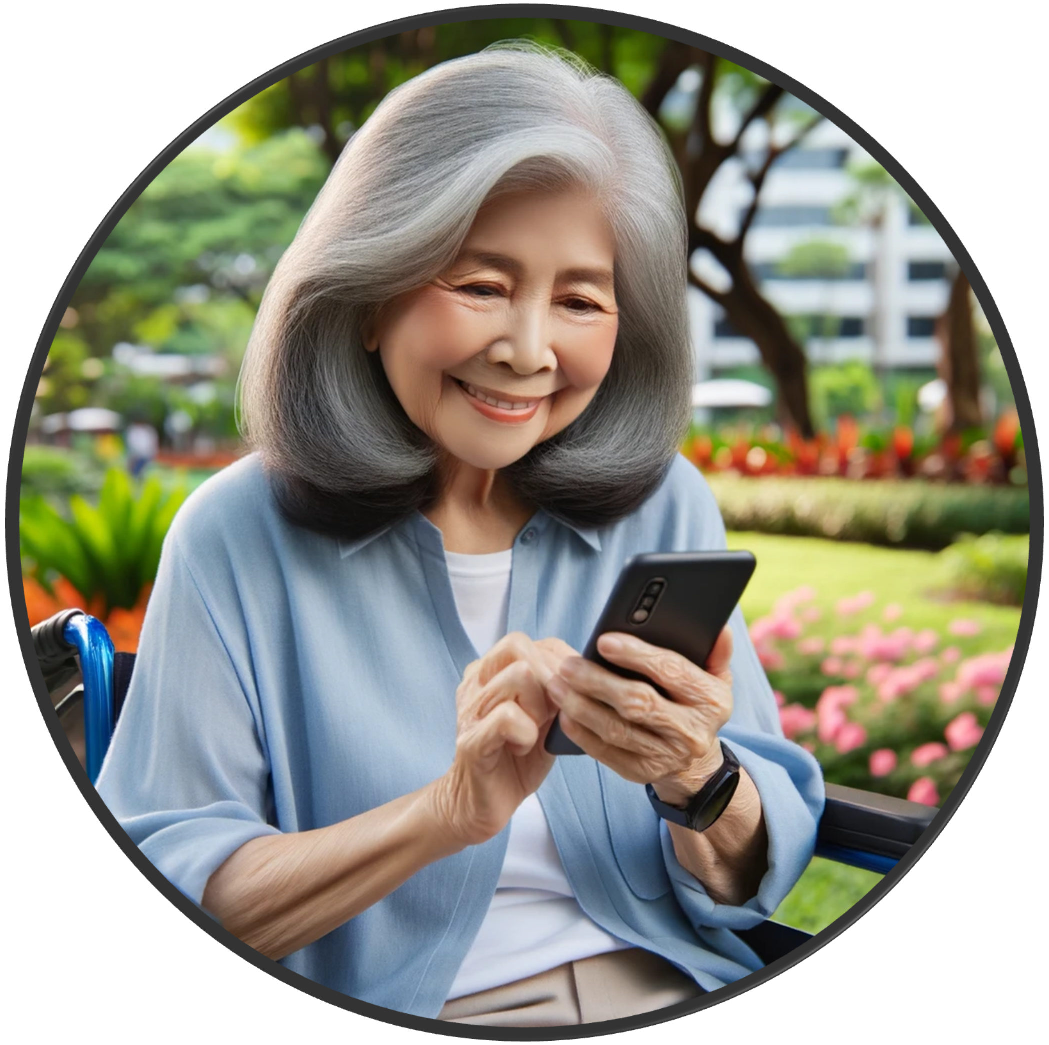

TOM

- Tom is 37 years old
- He is very **sporty** and loves both **long distance running** and local football team Arsenal
- Almost one year ago, he had a stroke and developed aphasia

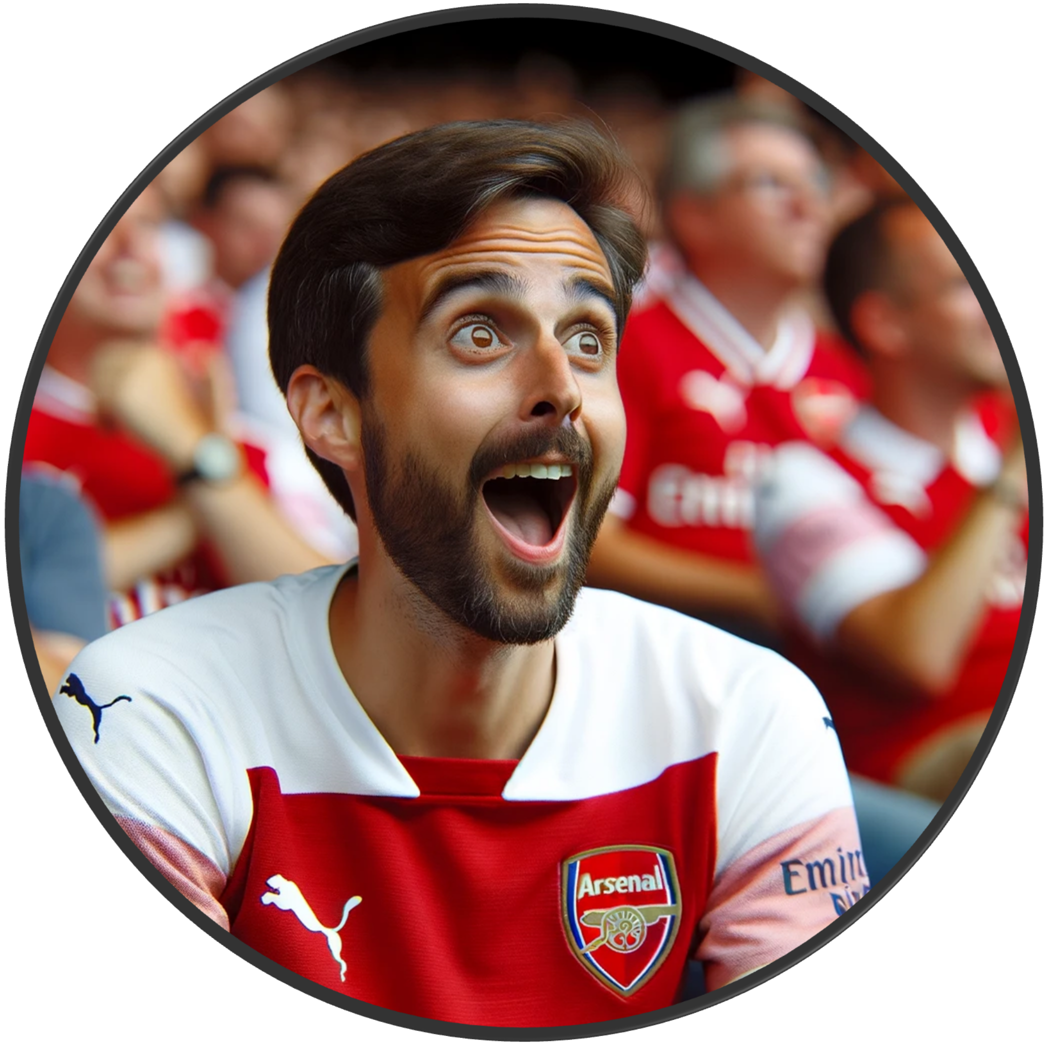

ALAN

- Alan is **65 years old**
- He was an **MP** in the 1990s and currently resides in West London
- He enjoys regular **holidays abroad** in France
- Following, Alan’s stroke 3 years ago, he is almost entirely **non-verbal with hemiplegia**

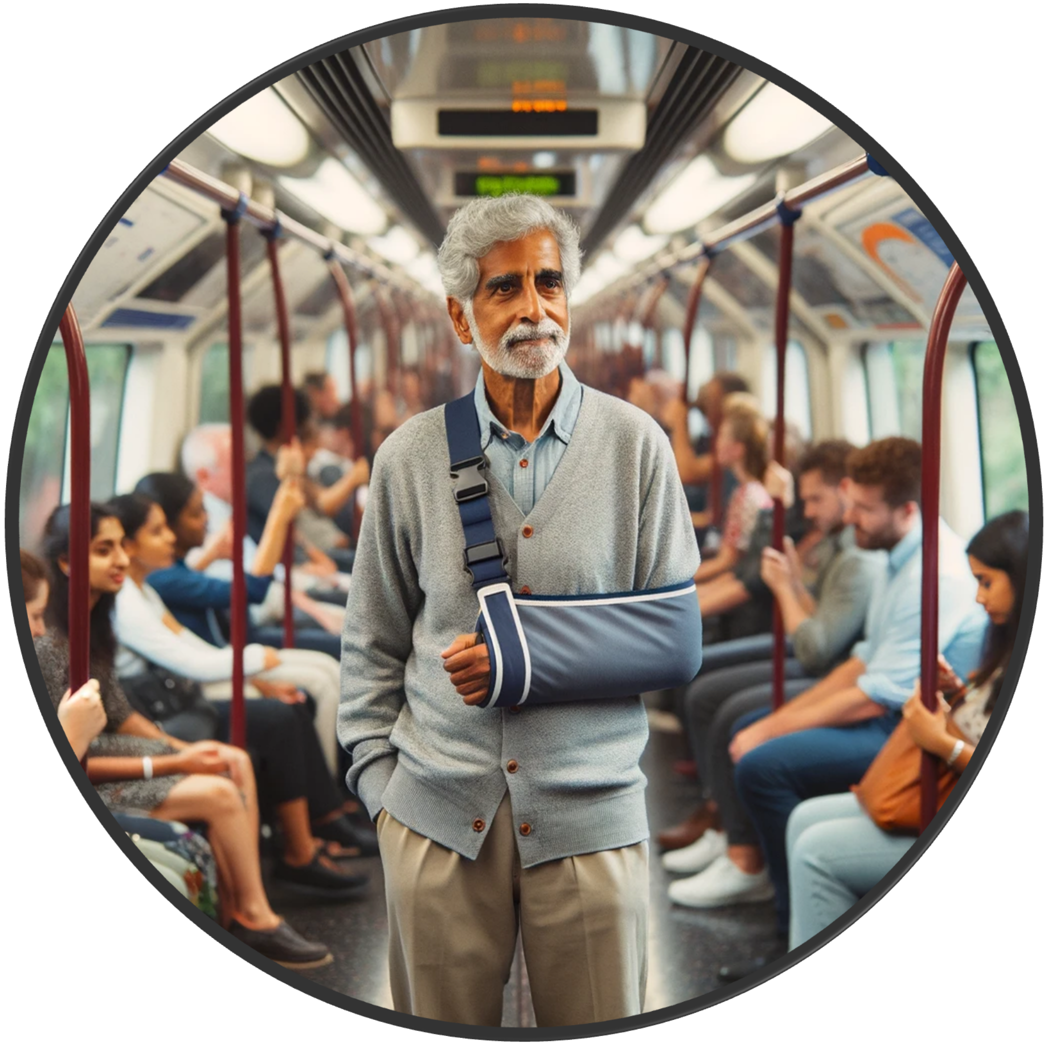

Supplement: Supplementary file 1 — Supplementary Information 1. [file 41598_2025_22253_MOESM1_ESM.zip › Supplementary/SM5.pdf]
