# Supplementary material for: Making smartglasses accessible: perspectives and prototypes from co-design with people with aphasia
Source: Sci Rep. 2025 Nov 3;15:38309. doi: 10.1038/s41598-025-22253-2 (PMC12583751; doi:10.1038/s41598-025-22253-2)
Supplement: Supplementary file 1 — Supplementary Information 1. [file 41598_2025_22253_MOESM1_ESM.zip › Supplementary/SM1.pdf]

Section and topic	Item	Reported on
1: Aim	*Report the aim of PPI in the study*<br><br>Smartglasses are poised to become a mainstream consumer technology in the near future. However, emerging technologies often fail to consider the accessibility needs of people with disabilities and older adults, heightening the risk of exclusion and marginalization. To address this gap, we conducted three exploratory co-design workshops with people living with aphasia (N=14), recruited as co-designers through the local charity Aphasia Re-Connect. The aim of this research was to examine and explore people with aphasia's perspectives on smartglasses, envision potential smartglass applications, and identify anticipated smartglass accessibility barriers.	Page 1, 3, Table 1, 2
2: Methods	*Provide a clear description of the methods used for PPI in the study*<br><br>We conducted three co-design workshops involving 14 people living with aphasia and 7 speech and language therapists (SLTs) to support the co-design of mixed reality (MR) smartglass technologies. The workshops, each lasting two and a half hours and spaced approximately one week apart, employed a range of accessible co-design methods. All sessions were audio- and video-recorded for qualitative analysis.<br><br>Workshop 1 explored co-designers' perspectives on diegetic smartglass prototypes featured in films and advertisements showcasing the latest MR technologies. Participants explored representations of smartglasses using curated video clips from films and advertising, chosen to illustrate a variety of form-factors, interaction styles, and use contexts. This activity was designed to prompt discussion about the potential uses, risks, and desirability of smartglass technologies. Specifically, these clips were sourced from both films and terrestrial advertising.<br><br>Workshop 2 focused on low-fidelity prototyping of smartglasses for video prompts, enabling co-designers to envision and prototype a range of MR smartglass technologies in an accessible manner. Using video prompts that depicted fictional characters with aphasia encountering real-life accessibility barriers, participants imagined and prototyped smartglass solutions. Initially, lensless cardboard cutout glasses helped co-designers reflect on different smartglass form factors, aesthetics, and social acceptability. Additionally, craft materials (e.g., fabric, card, string) and constructor straws enabled co-designers to prototype MR assets, including their 3D size and	Pages 2-5, Figure 3, 4, 7, 8, SM2, SM3, SM4 and SM5

	spatial proportions. This approach enabled people with aphasia to physically and visually co-design ideas in an accessible, hands-on way.<br><br>Finally, for Workshop 3 people with aphasia freely evaluated and tested a smartglass HoloLens HMD application built by the research team to support mobility and communication in public settings. Participants interacted with a functional HoloLens prototype developed by the research team to support mobility and communication in public settings. Supported by accessible handouts and one-on-one facilitation from a researcher or SLT, participants tested the device and provided feedback through questionnaires and group discussion. Insights gathered informed future smartglass design refinements.<br><br>PPI occurred throughout the entire research process: participants were not only study subjects but active collaborators whose lived experiences directly shaped the co-design and evaluation of the smartglass technologies.	
3: Results	*Outcomes – Report the results of PPI in the study, including both positive and negative outcomes*<br><br>PPI through co-design workshops contributed to the study in several substantive ways. Initially, engaging people with aphasia allowed us to understand how they envision smartglasses supporting their independent living. In particular, participants desired general applications (i.e., videoconferencing, navigation, object detection and information retrieval) plus smartglass supports for aphasia (i.e., reading text, spatial audio, dialogue support and memory aids).<br><br>Furthermore, co-designers with aphasia anticipated a raft of accessibility barriers to smartglasses, drawing directly from their lived experiences. Participants raised smartglass interactional concerns (i.e., physical gesture difficulties, cognitive load, surveillance concerns) and hardware challenges (i.e., antisocial form factor, discomfort, expensive costs and low-confidence/anxiety).<br><br>In addition to sharing qualitative insights through discussions, co-designers actively participated in envisioning and constructing 10 low-fidelity smartglass prototypes. These low-fidelity designs explored diverse form factors, personal style, visual media content, navigation and audio interaction – demonstrating a broad spectrum of user-driven innovation.<br><br>In the final workshop, participants evaluated a high-fidelity smartglass application developed by the research team using a HoloLens 2 HMD headset. This prototype was designed to support people with aphasia's communication, mobility, and navigation	Pages 5-11, Figures 2-8

	across public settings. Participants gave both quantitative feedback via questionnaires and qualitative feedback through facilitated group discussions. On the whole, the evaluated high-fidelity prototype was mixed – with most generally considering the device a 'work in progress'.	
4: Discussion	*Outcomes – Comment on the extent to which PPI influenced the study overall. Describe positive and negative effects.*<br><br>Given that participatory design methods were embedded throughout the three workshops, PPI played a central role in shaping the study outcomes.<br><br>In the early co-design sessions, participants with aphasia envisioned a wide range of immersive smartglass applications to support independence, mobility and communication. These included features such as videoconferencing, obstacle detection, navigation assistance and context-aware information delivery (e.g., stair-free route guidance). Participants emphasized the importance of timely, situational smartglass support – especially during high-stress mobility and communication scenarios. A further notable outcome was the emphasis on smartglass aesthetics. Co-designers expressed a strong desire for smartglasses to be not only functional but also personally expressive. They favoured bold, colourful designs that reflected their individual style.<br><br>Many of the anticipated barriers to smartglasses were validated in the evaluation of a high-fidelity prototype. The bulky and conspicuous form factor of the HoloLens HMD was seen as unsuitable for public use, and the gesture-based interaction model proved largely inaccessible. Fine motor gestures – such as pinching or interacting with virtual buttons lacking tactile feedback – were difficult or impossible for many participants. These challenges underscore the need for alternative, tangible interaction methods, such as physical controllers or external devices, in future smartglass designs.<br><br>Overall, PPI and co-design played a foundational role throughout the research. Revealing smartglasss accessibility challenges, contributing innovative co-design ideas, and evaluating real-world usability limitations. While technical constraints of current smartglass platforms limited implementation, the participatory approach ensured that people with aphasia can directly inform the continued development of smartglass technologies. This underscores the importance of involving people with disabilities early in the design process to ensure more inclusive and effective technologies.	Pages 11-13, Figures 2-8

5: Reflections	*Critical perspective – Comment critically on the study, reflecting on the things that went well and those that did not, so others can learn from this experience*<br><br>The study benefited greatly from the active and sustained involvement of people with aphasia, who self-reported high levels of enjoyment and engagement across all three co-design workshops. Their participation yielded a rich diversity of creative ideas and meaningful contributions to both low- and high-fidelity smartglass prototypes.<br><br>Eventually, some of the key features of the high-fidelity HoloLens smartglass prototype (e.g., verbal hand prompts) were well received, and participants proposed several further promising concepts for future smartglass development (e.g., real-time contextual subtitles). Many participants with hemiplegia were even able to operate the high-fidelity smartglass prototype with just one hand for interaction.<br><br>However, the study also revealed significant smartglass limitations that warrant further reflection. The high-fidelity prototype received mixed feedback and was consistently described as a "work in progress". Gesture-based controls proved especially problematic – physically demanding, cognitively taxing, and lacking in tactile feedback. These issues not only impacted usability but also led to frustration among participants. Public use of the smartglasses added a social barrier; gesturing in public was often experienced as embarrassing or socially awkward. Additionally, the physical strain of prolonged smartglass usage led to fatigue, underscoring the need for more ergonomic design. Concerns were also raised about surveillance and privacy, particularly due to the presence of multiple smartglass cameras.<br><br>These challenges highlight the importance of ongoing iteration, inclusive testing, and consideration of both functional and social aspects of smartglasses and assistive technologies. While participatory design enabled critical insights to emerge early, the technical constraints of current smartglass platforms limited the ability to fully realize participants' visions. Future research should look to continue to align emerging technologies with the needs and preferences of people living with disabilities.	Pages 11-13
