# Supplementary material for: Making smartglasses accessible: perspectives and prototypes from co-design with people with aphasia
Source: Sci Rep. 2025 Nov 3;15:38309. doi: 10.1038/s41598-025-22253-2 (PMC12583751; doi:10.1038/s41598-025-22253-2)
Supplement: Supplementary file 1 — Supplementary Information 1. [file 41598_2025_22253_MOESM1_ESM.zip › Supplementary/SM-Descriptions.pdf]

Supplementary file descriptions:

SM1.pdf - A GRIPP2 Short Form checklist was used to report patient and public involvement, in line with best practices in participatory research.

SM2.pdf - Three images of participants engaging with scenario grid activities during Workshop 1. The images show three participants completing co-design tasks related to imagined smartglass use, including their opinions, contexts of use, communication challenges, expected social interactions, and potential affordances or solutions offered by smartglass technologies.

SM3.pdf - Series of images from Workshop 2. At the top, co-designers using various materials – such as plastic glass frames, paper, pens, scissors, and constructor straws – to prototype smartglasses and MR assets. At the bottom, an image of the assembled design materials before the workshop.

SM4 - 3 of the 5 AI video prompts played to participants during workshop 2 used to ideate low-fidelity AAC prototypes. The video prompts have been adapted to remove AI-generated imagery in accordance with editorial guidelines.

SM4.srt - Captions file for SM4 video.

SM5.pdf - Tangible and accessible character cards used to introduce video prompt characters during Workshop 2.

SM6.pdf - Series of images showing co-designers testing and evaluating the high-fidelity HoloLens HMD in Workshop 3. From top left to right: co-designers testing both menus and evaluating the discreetness of gestures in public. From bottom left to right: co-designers adjusting scalable maps, pinching, moving, and scaling assets, and an internal view of the application's features fully deployed within the community centre.

SM7 - Demo video of Hololens MR headset application developed, evaluated and tested by the participants in Workshop 3.

SM7.srt - Captions file for SM5 video.

SM8.pdf - Low-fidelity smartglass prototypes built by co-designers in Workshop 2 categorized into three envisioned aspects: (1) form factors, (2) visual media, and (3) audio interactions.
