# Supplementary material for: Making smartglasses accessible: perspectives and prototypes from co-design with people with aphasia
Source: Sci Rep. 2025 Nov 3;15:38309. doi: 10.1038/s41598-025-22253-2 (PMC12583751; doi:10.1038/s41598-025-22253-2)
Supplement: Supplementary file 1 — Supplementary Information 1. [file 41598_2025_22253_MOESM1_ESM.zip › Supplementary/SM8.pdf]

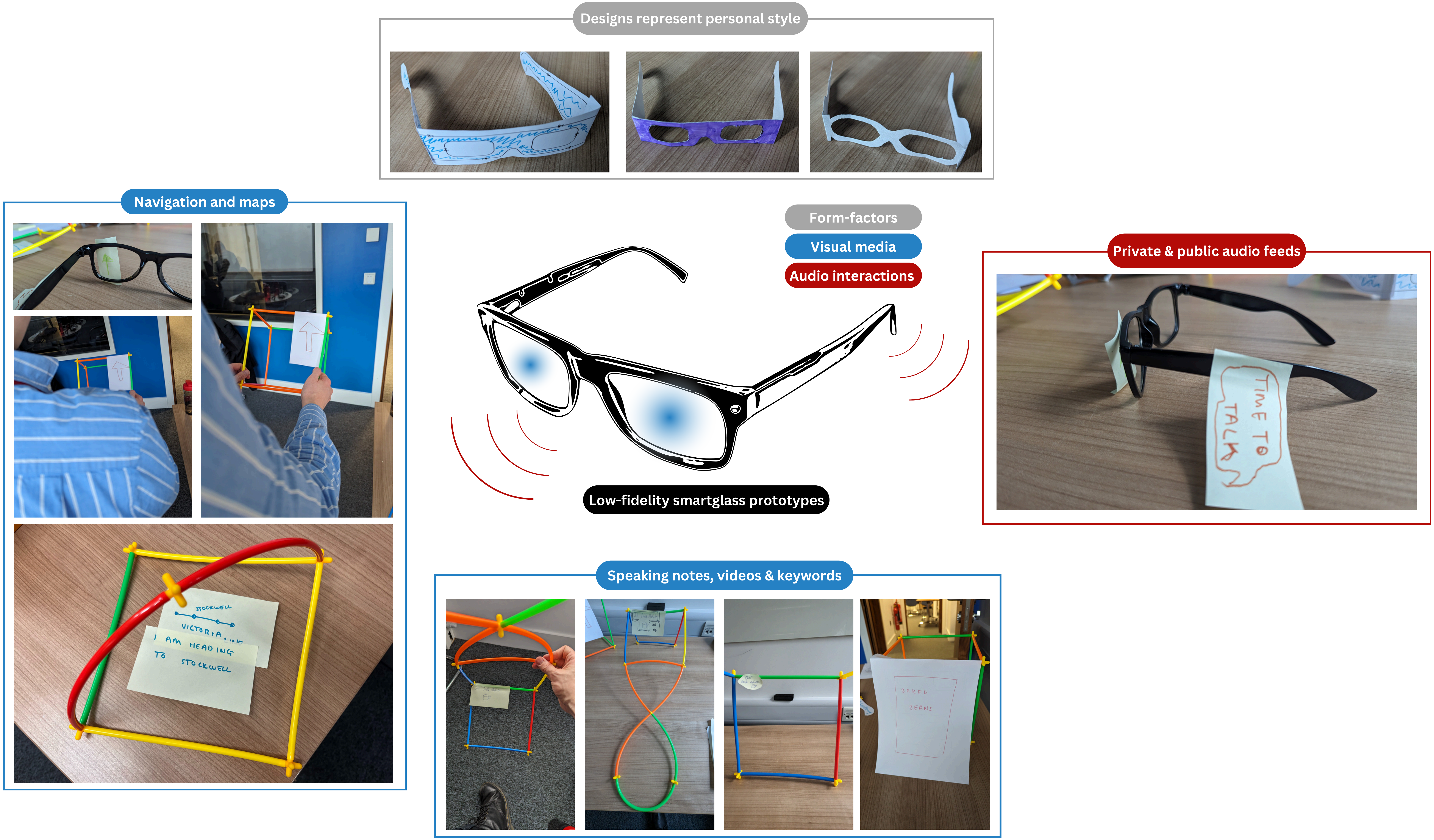
Designs represent personal style
Navigation and maps
Form-factors
Visual media
Audio interactions
Private & public audio feeds
Low-fidelity smartglass prototypes
Speaking notes, videos & keywords
STOCKWELL
VICTORIA LINE
I AM HEADING TO STOCKWELL
TIME TO TALK
BAKED BEANS
