# Supplementary figures and images for: Making smartglasses accessible: perspectives and prototypes from co-design with people with aphasia
Source: Sci Rep. 2025 Nov 3;15:38309. doi: 10.1038/s41598-025-22253-2 (PMC12583751; doi:10.1038/s41598-025-22253-2)

Participants low-fidelity prototype

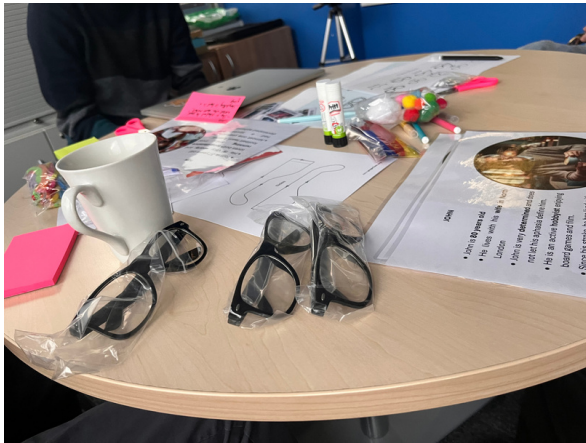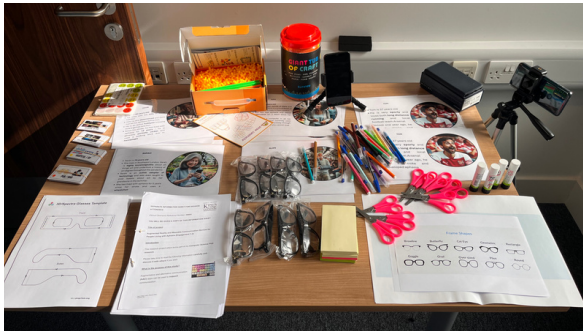

Design materials for low-fidelity prototyping

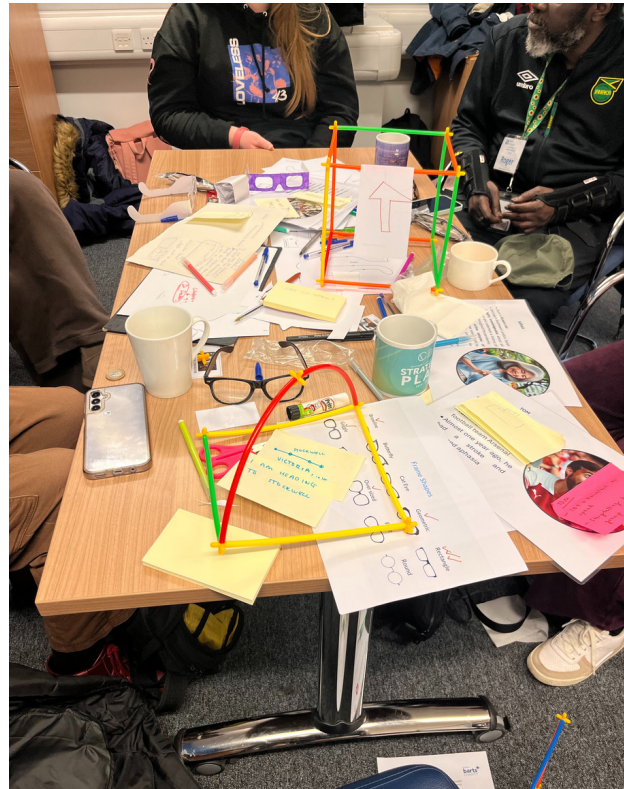

Supplement: Supplementary file 1 — Supplementary Information 1. [file 41598_2025_22253_MOESM1_ESM.zip › Supplementary/SM3.pdf]

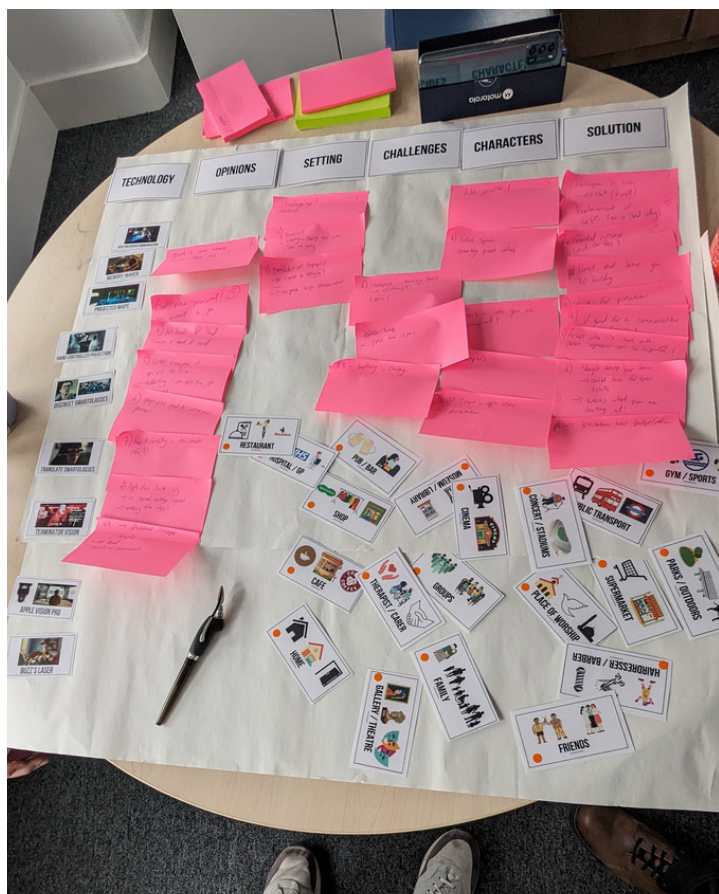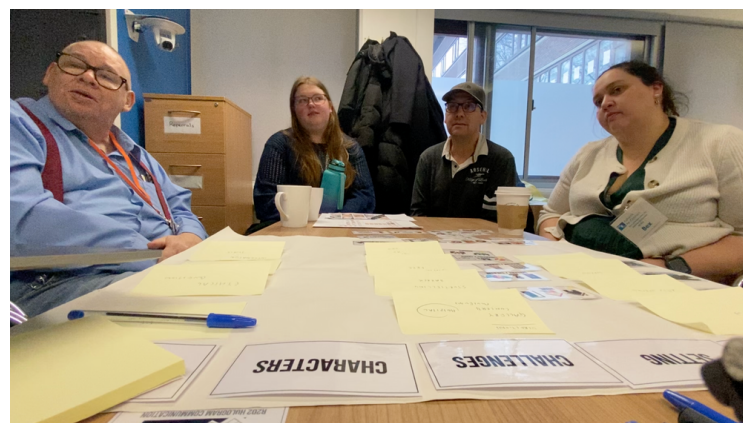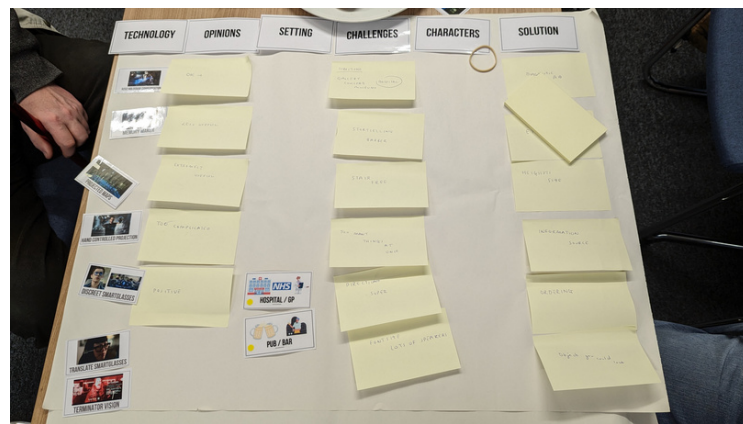

Participants complete Talking Mat Scenario Grids

Supplement: Supplementary file 1 — Supplementary Information 1. [file 41598_2025_22253_MOESM1_ESM.zip › Supplementary/SM2.pdf]
